# *Scn2a* severe hypomorphic mutation decreases excitatory synaptic input and causes autism-associated behaviors

**DOI:** 10.1172/jci.insight.150698

**Published:** 2021-08-09

**Authors:** Hong-Gang Wang, Charlotte C. Bavley, Anfei Li, Rebecca M. Jones, Jonathan Hackett, Yared Bayleyen, Francis S. Lee, Anjali M. Rajadhyaksha, Geoffrey S. Pitt

**Affiliations:** 1 Cardiovascular Research Institute,; 2 Feil Family Brain and Mind Research Institute, and; 3 Pediatric Neurology, Department of Pediatrics, Weill Cornell Medicine, New York, New York, USA.; 4 Weill Cornell Medicine, Center for Autism and the Developing Brain, White Plains, New York, USA.; 5 Weill Cornell Autism Research Program and; 6 Sackler Institute for Developmental Psychobiology, Department of Psychiatry, Weill Cornell Medicine, New York, New York, USA.

**Keywords:** Neuroscience, Ion channels, Mouse models

## Abstract

*SCN2A*, encoding the neuronal voltage-gated Na^+^ channel Na_V_1.2, is one of the most commonly affected loci linked to autism spectrum disorders (ASDs). Most ASD-associated mutations in *SCN2A* are loss-of-function mutations, but studies examining how such mutations affect neuronal function and whether *Scn2a* mutant mice display ASD endophenotypes have been inconsistent. We generated a protein truncation variant *Scn2a* mouse model (*Scn2a*^Δ1898/+^**) by CRISPR that eliminates the Na_V_1.2 channel’s distal intracellular C-terminal domain, and we analyzed the molecular and cellular consequences of this variant in a heterologous expression system, in neuronal culture, in brain slices, and in vivo. We also analyzed multiple behaviors in WT and *Scn2a*^Δ1898/+^** mice and correlated behaviors with clinical data obtained in human subjects with *SCN2A* variants. Expression of the Na_V_1.2 mutant in a heterologous expression system revealed decreased Na_V_1.2 channel function, and cultured pyramidal neurons isolated from *Scn2a*^Δ1898/+^** forebrain showed correspondingly reduced voltage-gated Na^+^ channel currents without compensation from other CNS voltage-gated Na^+^ channels. Na^+^ currents in inhibitory neurons were unaffected. Consistent with loss of voltage-gated Na^+^ channel currents, *Scn2a*^Δ1898/+^** pyramidal neurons displayed reduced excitability in forebrain neuronal culture and reduced excitatory synaptic input onto the pyramidal neurons in brain slices. *Scn2a*^Δ1898/+^** mice displayed several behavioral abnormalities, including abnormal social interactions that reflect behavior observed in humans with ASD and with harboring loss-of-function *SCN2A* variants. This model and its cellular electrophysiological characterizations provide a framework for tracing how a *SCN2A* loss-of-function variant leads to cellular defects that result in ASD-associated behaviors.

## Graphical abstract

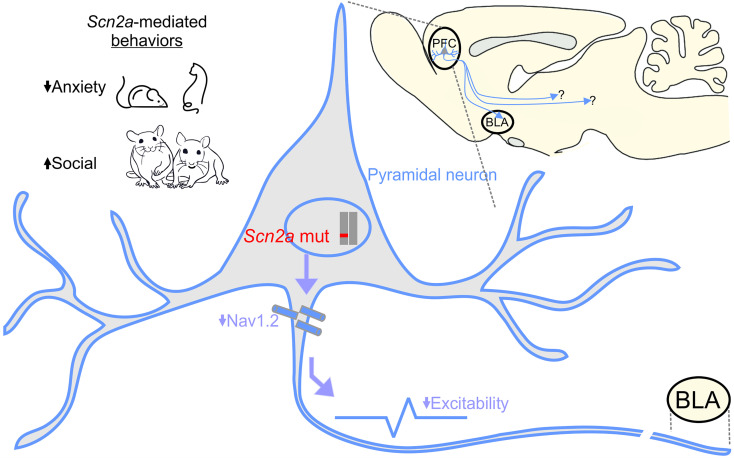

## Introduction

Within the CNS, voltage-gated Na^+^ (Na_V_) channels such as the *SCN2A*-encoded Na_V_1.2 initiate action potentials (APs) and are, thus, fundamental to defining neuronal excitability. In addition to Na_V_1.2, which is found in excitatory neurons and a small set of inhibitory interneurons (1), the major brain Na_V_ channels are *SCN1A*-encoded Na_V_1.1 (expressed mainly in inhibitory neurons), *SCN3A*-encoded Na_V_1.3 (expressed in embryonic neurons), and *SCN8A*-encoded Na_V_1.6 (found in excitatory and inhibitory neurons). Most mature excitatory CNS neurons express Na_V_1.2 and Na_V_1.6, and these Na_V_ channels confer distinct features that tweak electrical activity and contribute to the defining features of AP initiation and conduction in different types of neurons. During early brain development, Na_V_1.2 is the dominant Na^+^ channel expressed in excitatory neurons, where it predominantly localizes to the axon initial segment (2, 3). Because of this preferential expression of Na_V_1.2 during the vulnerable developmental period when most autism spectrum disorder–associated (ASD-associated) mutations exert their influence (4), Na_V_1.2 is well positioned to exert a potent effect on AP initiation and conduction, thereby influencing neuronal excitability and activity-dependent development in the maturing brain.

Next-generation sequencing studies of de novo mutations in subjects with ASD have consistently identified *SCN2A* as one of the most commonly associated loci (5–7), and *SCN2A* is also among the top loci identified with postzygotic mutations associated with ASD (8). Variants in *SCN2A* have also been linked to several other neurological disorders, including epilepsy and intellectual disability (9). Analyses in heterologous expression systems of several disease-associated *SCN2A* variants found that ASD-associated mutations generally cause channel loss-of-function effects (often because of protein truncation variants), while epilepsy-associated mutations reveal various biophysical gain-of-function effects (increased Na^+^ influx) (10).

Recent examinations of *Scn2a* heterozygous KO (*Scn2a^+/–^)* mice showed that, in addition to the critical role in the axonal excitability in early development, Na_V_1.2 is crucial to AP backpropagation, dendritic excitability, synaptic transmission, and synaptic plasticity in mature pyramidal neurons (11, 12). These *Scn2a^+/–^* mice revealed a decrease in learning and memory (13), but other behavioral consequences, especially those associated with autistic-like behaviors, were highly variable and often inconsistent (11, 12, 14, 15). Therefore, the specific contribution of Na_V_1.2 dysfunction to ASD endophenotypes has not been determined.

A perturbation of the delicate balance between neuronal excitation and inhibition (E/I balance) has been implicated in a range of neurodevelopmental disorders (4), and a leading hypothesis is that ASD results from an E/I imbalance in developing circuits during the critical neonatal period (16, 17). By exploiting mice with a Na_V_1.2 protein truncation variant (*Scn2a*^Δ1898/+^**) leading to severe reduction in channel function, we investigated the consequences on neuronal cellular properties and synaptic transmission to examine effects on E/I balance and ASD-associated behaviors. We found that severe reduction of Na_V_1.2 function reduced neuronal excitability in cultured forebrain pyramidal neurons isolated from *Scn2a*^Δ1898/+^** forebrains, and it decreased excitatory synaptic input to pyramidal neurons in the medial prefrontal cortex (mPFC) and basolateral amygdala (BLA) in acute brain slice from adult *Scn2a*^Δ1898/+^** mice. *Scn2a*^Δ1898/+^** mice displayed several behavioral abnormalities, including enhanced sociability and lack of typical social habituation, consistent with behavior in humans with loss-of-function variants in *SCN2A*. Together, this model and the accompanying cellular electrophysiological characterizations provide a framework for tracing a *SCN2A* loss-of-function variant to cellular and synaptic defects and the resulting altered E/I balance that is associated with ASD-associated behaviors.

## Results

### Scn2a severe hypomorph mouse model.

**Figure 1:**
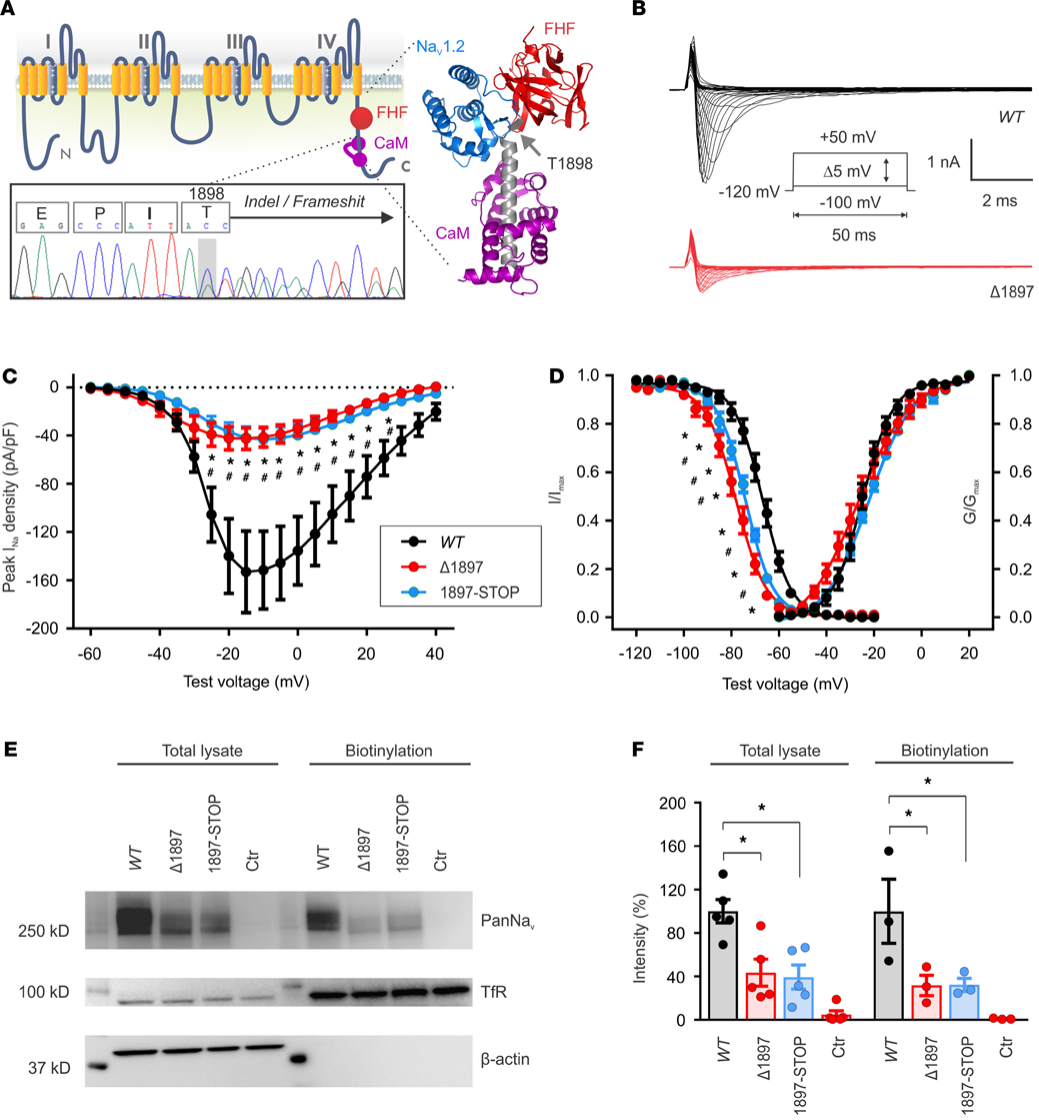
Na_V_1.2^Δ1897^ channels display reduced peak Na^+^ current density in transfected cells. (**A**) Schematic of the Na_V_1.2 pore-forming α subunit. The bottom inset showing genomic DNA sequencing, which demonstrates the T1898N frameshift in one of the alleles in *Scn2a*^Δ1898/+^** mice. The right inset shows the location of T1898 on the crystal structure (Protein Data Bank entry: 4JPZ) of the ternary complex of the Na_V_1.2 C-terminal domain (CTD, blue; truncated helix shown in gray), FGF13 (FHF, red), and calmodulin (purple). The arrow indicates the location of T1898. (**B**) Exemplar current traces for Na_V_1.2^WT^ and the frameshifted/truncated Na_V_1.2^Δ1897^ channel (p. T1897NsfX27, equivalent to T1898 in mice) expressed in HEK293 cells. (**C**) Peak current density-voltage relationships for Na_V_1.2^WT^ (*n =* 11), Na_V_1.2^Δ1897^ (Δ1897, *n =* 11), and a Na_V_1.2 with a stop codon inserted at T1897 (1897–STOP, *n =* 12). Asterisks represent 2-way ANOVA followed by Dunnett’s multiple-comparison test. Peak I_Na_ density × mutation, F_(40, 620)_ = 9.732, *P <* 0.0001. (**D**) Steady-state inactivation (I/I_max_) (WT, *n =* 15; Δ1897, *n =* 11; 1897–STOP, *n =* 12) and activation (G/G_max_) relationships for the 3 channels. Asterisks represent 2-way ANOVA followed by Dunnett’s multiple-comparison test. I/I_max_ × mutation, F_(40, 700)_ = 12.34, *P <* 0.0001. (**E**) Exemplar immunoblot of whole cell lysates or the biotinylated surface fraction from HEK293 cells expressing the 3 channels. Transferrin receptor (TfR) and actin represent a membrane and cytoplasmic marker, respectively, that demonstrate successful separation of the biotinylated membrane fraction. Molecular weight markers are shown on the left. See complete unedited blots in the supplemental material. (**F**) Quantification of intensities (relative to WT) from immunoblots (total lysate, *n =* 5; biotinylation, *n =* 3). Asterisks represent 1-way ANOVA followed by Dunnett’s multiple-comparison test. Total lysate, F_(3, 16)_ = 15.4, *P <* 0.0001; Na_V_1.2^WT^ versus Na_V_1.2^Δ1897^, *P =* 0.0029; Na_V_1.2^WT^ versus 1897–STOP, *P =* 0.0016. Biotinylation, F_(3, 8)_ = 6.963, *P =* 0.01; Na_V_1.2^WT^ versus Δ1897, *P =* 0.04; Na_V_1.2^WT^ versus 1897–STOP, *P =* 0.04.

Like all other Na_V_ channel α subunits, the *SCN2A*-encoded Na_V_1.2 has pseudotetrameric repeats of 6 transmembrane segments (Figure 1A) joined by intracellular loops and flanked by cytoplasmic N- and C-termini. The *SCN2A* transcript contains 27 exons, the last of which is the largest and encodes approximately 400 amino acids that include the fourth tetrameric repeat and the C-terminus. In a compendium accompanying a recent study identifying risk genes with autism and developmental disability biases, 7 of 27 *SCN2A* mutations were within this exon (consistent with the length of polypeptide as a fraction of the overall length of the channel), 5 of which induced a frameshift and protein truncation (7). Overall, 8 of 27 *SCN2A* mutations in that compendium had a frameshift, so frameshifts may be overrepresented in this final exon. While generating a single-point mutation mouse knock-in model of an *SCN2A* disease mutation using CRISPR/Cas9 (to be reported separately), we also obtained an indel that produced a frameshift after T1898 in the Na_V_1.2 C-terminus and a subsequent stop codon following 26 potentially novel amino acids (p.T1898NfsX27), as shown in Figure 1A and Supplemental Figure 1 (supplemental material available online with this article; https://doi.org/10.1172/jci.insight.150698DS1). Truncation of Na_V_1.2 at T1898 eliminates the binding site for the channel auxiliary subunit calmodulin (Figure 1A), which regulates the persistent Na^+^ current (18, 19). Because the indel fit a pattern (protein truncation variant in the final exon) common to multiple ASD-associated mutations in *SCN2A*, we chose to analyze the consequences of this allele (*Scn2a*^Δ1898^**) further.

Heterozygous *Scn2a*^Δ1898/+^** mice were viable and fertile, but we never obtained homozygous *Scn2a*^Δ*1898/*Δ1898^** mice at weaning from heterozygous crosses (*Scn2a*^Δ1898/+^**, *n =* 24; WT, *n =* 13; *Scn2a*^Δ*1898/*Δ1898^**, *n =* 0; χ^2^ = 12.4, *P <* 0.01). Since *Scn2a^–/–^* display perinatal mortality due to hypoxia from absent respirations (20), we suspect that the *Scn2a*^Δ1898^** is a severe hypomorphic allele. The approximately 2:1 ratio of WT to *Scn2a*^Δ1898/+^** heterozygotes suggests that *Scn2a* haploinsufficiency does not affect survival, as previously observed (20).

To determine the consequences to Na_V_1.2 Na^+^ currents, we expressed a frameshifted human Na_V_1.2 channel (Na_V_1.2^Δ1897^, equivalent to Na_V_1.2^Δ1898^ in mice) in HEK293 cells and recorded Na^+^ currents by whole-cell voltage clamp. Compared with cells expressing Na_V_1.2^WT^ channels, cells with Na_V_1.2^Δ1897^ channels showed markedly decreased Na^+^ current density (Figure 1, B and C). We also measured currents from a human Na_V_1.2 in which we inserted a stop codon after T1897 (Na_V_1.2^1897–STOP^), thereby eliminating the alternative 26 amino acids generated by the frameshift, and we observed a similar reduction in current density (Figure 1C). In addition to reducing current density, we found that both the frameshift and the truncation decreased channel availability, as indicated by the leftward shift of the channel steady-state inactivation curves (Figure 1D). The voltage at half-maximal availability (V_1/2_) for steady-state inactivation was –77.9 mV and –74.0 mV for Na_V_1.2^Δ1897^ and Na_V_1.2^1897–STOP^, respectively — a significant hyperpolarizing shift from the –67.7 mV for the WT channel.

To examine whether the reduced Na^+^ current resulted from less Na_V_1.2^Δ1897^ or Na_V_1.2^1897–STOP^ protein inserted into the plasma membrane, we performed surface biotinylation, streptavidin pull down, and quantification of surface Na_V_1.2 by immunoblot. Figure 1, E and F, shows that, compared with Na_V_1.2^WT^, total Na_V_1.2^Δ1897^ or Na_V_1.2^1897–STOP^ protein was approximately 40% of Na_V_1.2^WT^ and that the amount of Na_V_1.2^Δ1897^ or Na_V_1.2^1897–STOP^ inserted into the plasma membrane was similarly reduced to approximately 32% of Na_V_1.2^WT^. These data suggest that the frameshift/truncation led to decreased protein synthesis or increased degradation, but did not exert significant effects on trafficking to the plasma membrane. Since Na_V_1.2^1897^ and Na_V_1.2^1897–STOP^ displayed nearly identical biophysical effects and showed similar degrees of protein instability, we conclude that the additional 26 amino acids generated by the frameshift do not overtly influence Na_V_1.2 channel function. Thus, Na_V_1.2^Δ1897^ behaved as a mutant channel with severely reduced function. These heterologous system data suggest that *Scn2a*^Δ1898/+^** mice represented an excellent platform to investigate loss-of-function *Scn2a-*associated ASD in an animal model.

**Figure 2:**
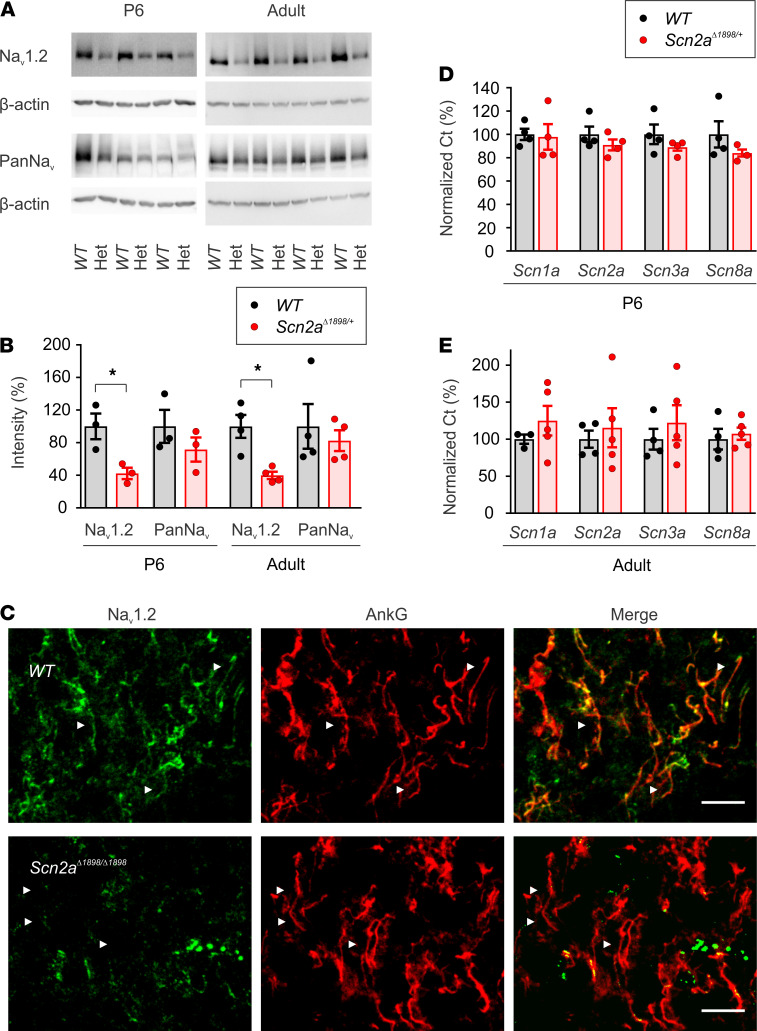
Cortices from *Scn2a^Δ1898/+^* mice have less total and Na_V_1.2 Na_V_ channels. (**A**) Immunoblots for Na_V_1.2 (anti-Na_V_1.2, recognizes amino acids 1882–2005) or total voltage-gated Na^+^ channel (PanNa_V_) in brain cortex lysates from P6 (*n =* 3) or adult (~5 month, *n =* 4) WT or *Scn2a*^Δ1898/+^** (Het) mice. Molecular weights are shown on the left. See complete unedited blots in the supplemental material. (**B**) β-Actin serves as a loading control and for normalization in **B**, which shows the quantification of Na_V_1.2 and total Na^+^ channels as in **A**, normalized to WT. Asterisks represent unpaired *t* test, t_(4)_ = 3.348, *P =* 0.03 for P6; t_(6)_ = 4.07, *P =* 0.007 for adult. (**C**) IHC of layer 2/3 in cortex from P0.5 mice stained with anti-Na_V_1.2 and anti–ankyrin G (AnkG) antibodies. The arrows indicate axonal Na_V_1.2 or AnkG. Scale bar: 10 μm. (**D** and **E**) Relative (normalized to GAPDH) CNS Na^+^ channel transcripts quantified by qPCR in P3–P6 (*n =* 4) and adult (~5 month; WT, *n =* 4; *Scn2a*^Δ1898/+^**, *n =* 5) cortex, respectively.

To test whether the mutation also led to reduced Na_V_1.2 protein in vivo as observed in HEK293 cells, we analyzed the amount of Na_V_1.2 protein in lysates from cortex isolated from *Scn2a*^Δ1898/+^** mice and their WT littermates. We generated lysates from P6 neonates, a stage when Na_V_1.2 is the dominant Na_V_ channel in excitable cells before Na_V_1.6 replaces most Na_V_1.2 at the axon initial segment, and separately from adult (~5-month-old) animals. In both P6 and adult mice, we observed less Na_V_1.2 protein in *Scn2a*^Δ1898/+^** mice (reduced by ~60%), as detected by 2 different Na_V_1.2-specific antibodies targeting distinct epitopes in the channel (Figure 2, A and B, and Supplemental Figure 3). Using an antibody that recognizes all Na_V_ channels, we did not observe a statistically significant reduction in total Na^+^ channel protein in cortex lysates from P6 neonatal and adult *Scn2a*^Δ1898/+^** mice compared with their respective WT littermate controls (Figure 2, A and B, and Supplemental Figure 3). This is likely because Na_V_1.2 is just one of the CNS Na^+^ channels, and the loss of Na_V_1.2 from 1 *Scn2a* allele is insufficient to detect a significant reduction in the total Na^+^ channel protein pool.

Truncated proteins are often subjected to rapid degradation. To examine if Na_V_1.2^Δ1898^ channels were subject to degradation, we stained brain slices from newborn WT and *Scn2a*^Δ*1898/*Δ1898^** mice (which show perinatal lethality) for Na_V_1.2. Na_V_1.2 was abundantly present in the axonal initial segment (AIS) of WT mice, which was labeled with AIS-specific marker ankyrin G, in the layer 2/3 neocortex from WT mice, but was almost completely absent in the cortical tissue from *Scn2a*^Δ*1898/*Δ1898^** mice (Figure 2C). These data suggest that *Scn2a*^Δ1898^** encodes an unstable protein and functions as a severe hypomorphic allele.

Since total Na^+^ channel protein in brains from P6 and adult mice was not statistically different between genotypes, we ascertained whether the observed reduction in Na_V_1.2 protein led to a compensatory upregulation of other Na^+^ channel isoforms in the CNS. We performed quantitative PCR (qPCR) on mRNA isolated from the cortex of P3–P6 and adult mice and quantified the relative levels of transcripts for other Na^+^ channel isoforms. We did not observe genotype-specific differences between *Scn2a*^Δ1898/+^** and WT mice in the transcript levels for *Scn1a*, *Scn2a*, *Scn3a*, or *Scn8a* (Figure 2, D and E). The fact that there were no genotype-specific differences for *Scn2a* transcripts, specifically, suggests that the reduced Na_V_1.2 protein in *Scn2a*^Δ1898/+^** mice derives from Na_V_1.2^Δ1898^ protein instability and degradation.

### Neuronal cellular consequences of a Scn2a severe hypomorphic allele.

**Figure 3:**
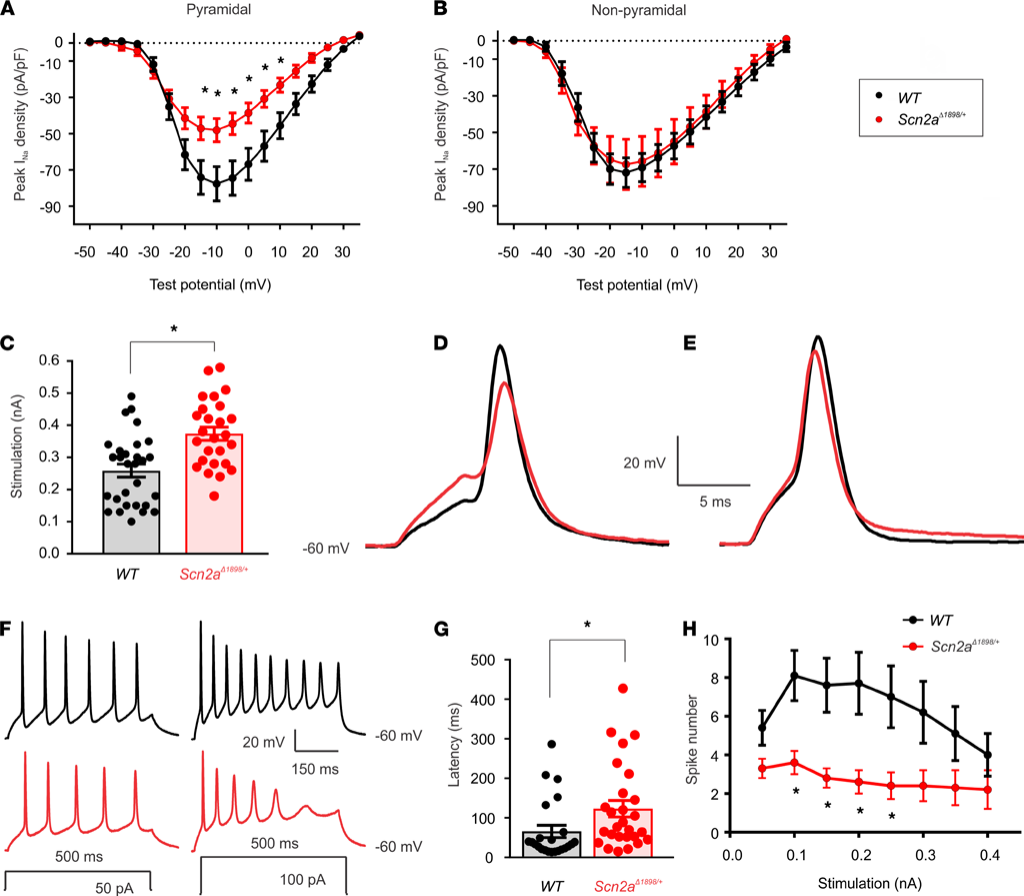
Cortical neurons from *Scn2a^Δ1898/+^* mice display reduced Na_V_ channel current and reduced excitability. (**A**) Current-voltage relationship for excitatory (pyramidal) neurons (WT, *n =* 14; *Scn2a*^Δ1898/+^**, *n =* 17). Asterisks represent 2-way ANOVA followed by Sidak’s multiple-comparison test. Peak I_Na_ density × genotype, F_(16, 464)_ = 6.73, *P <* 0.0001. (**B**) Current-voltage relationship for inhibitory (nonpyramidal) neurons (WT, *n =* 12; *Scn2a*^Δ1898/+^**, *n =* 12). (**C**) Stimulation threshold to elicit action potentials in cultured cortical neurons isolated from WT (*n =* 29) or *Scn2a*^Δ1898/+^** (*n =* 26). Asterisks represent unpaired *t* test, t_(53)_ = 3.961, *P =* 0.0002. (**D**) Exemplar action potentials from WT or *Scn2a*^Δ1898/+^** elicited at threshold stimulation. (**E**) Exemplar action potentials from WT or *Scn2a*^Δ1898/+^** elicited at a stimulation intensity (0.4 ± 0.03 nA and 0.6 ± 0.02 nA, for WT and *Scn2a*^Δ1898/+^**, respectively) that elicits the maximal amplitude. (**F**) Exemplar evoked action potential trains elicited from WT (*n =* 23) or *Scn2a*^Δ1898/+^** (*n =* 27) with 500 ms current injection of 50 pA or 100 pA. The resting membrane potential is indicated (bottom right). (**G**) Latency of first spike at minimum stimulation intensity. Asterisks represent unpaired *t* test, t_(48)_ = 2.135, *P =* 0.038. (**H**) The number of evoked action potentials for the indicated intensity of current injection. Asterisks represent 2-way ANOVA followed by Sidak’s multiple-comparison test. Spike number × genotype, F_(11, 528)_ = 6.693, *P <* 0.0001.

Having established the biophysical and biochemical consequences of the severe hypomorphic *Scn2a*^Δ1898^** allele, we queried the resulting cellular electrophysiological consequences. To best avoid the voltage clamp challenges of Na^+^ currents in brain slice recordings, we characterized the effect of *Scn2a*^Δ1898/+^** on neuronal cellular properties in young neuronal cultures, when neurons have fewer branches and Na_V_1.2 is the dominantly expressed Na^+^ channel. We isolated forebrain neurons from P1–P3 pups, cultured them for 5–7 days, and recorded total Na_V_ currents by whole-cell voltage clamp from pyramidal-shaped (excitatory) neurons (Supplemental Figure 2A). Peak current density was markedly reduced in neurons from *Scn2a*^Δ1898/+^** mice compared with their WT littermate controls. The I-V relationships shown in Figure 3A revealed a ~38% reduction in Na^+^ channel current density in *Scn2a*^Δ1898/+^** neurons. If we assume, based on the results in Figure 2, that this reduction represents the almost complete loss of 1 *Scn2a* allele, we calculate that Na_V_1.2 contributes ~75% of the total Na_V_ channel current in these young cortical pyramidal neurons. Because Na_V_1.2 has also been found in small set of inhibitory interneurons (1), we also analyzed the I-V relationships of Na^+^ currents in nonpyramidal shape neurons (Supplemental Figure 2B). As shown in Figure 3B, there was no difference in current density in *Scn2a*^Δ1898/+^** versus WT neurons. This is consistent with the reported results (21) showing reduced Na_V_1.2 currents in excitatory, but not inhibitory, neurons. Together, these data further support our interpretation that *Scn2a*^Δ1898/+^** mice represent a loss-of-function model.

As *Scn2a* is almost exclusively expressed in excitatory neurons, and since Na^+^ channels drive APs, we characterized APs elicited in the cultured forebrain pyramidal neurons in which we identified the reduction in total Na^+^ currents for *Scn2a*^Δ1898/+^** mice. We first measured the stimulus threshold at which APs were elicited and found that the threshold to elicit APs in cultured *Scn2a*^Δ1898/+^** pyramidal cortical neurons was significantly higher than that needed in WT neurons (Figure 3C). Moreover, the AP shape (Figure 3D) and kinetic parameters (Table 1) differed. Although our qPCR data (Figure 2, D and E) demonstrate the absence of transcriptional compensation from other CNS Na_V_ channels, we cannot exclude compensatory changes from other Na^+^ channels inserted into specific cellular locations normally dominated by Na_V_1.2, nor by other, non-Na^+^ channel ionic currents that underlie the AP. We attempted to assess the compensation from non-Na^+^ channel ionic currents by analyzing APs elicited by a strong stimulus designed to overcome the relative Na_V_1.2 channel deficit in *Scn2a*^Δ1898/+^** neurons. For a stimulus that elicited a maximal amplitude AP, we no longer observed a difference in the AP amplitude between *Scn2a*^Δ1898/+^** and WT littermate control neurons, suggesting that the reduced AP amplitude in *Scn2a*^Δ1898/+^** neurons was derived mainly from the reduced Na^+^ current amplitude (Figure 3E and Table 2). Furthermore, we observed a marked decrease in the number of spikes elicited from *Scn2a*^Δ1898/+^** neurons across a range of stimulation intensities and, consistent with the reduced overall Na^+^ channel current amplitude in *Scn2a*^Δ1898/+^** neurons, the latency to first spike was longer than in WT neurons (Figure 3, F–H). Together, these data show that the *Scn2a*^Δ1898/+^** pyramidal neurons were less excitable than WT neurons.

### Behavioral consequences of a Scn2a severe hypomorphic allele.

**Figure 4:**
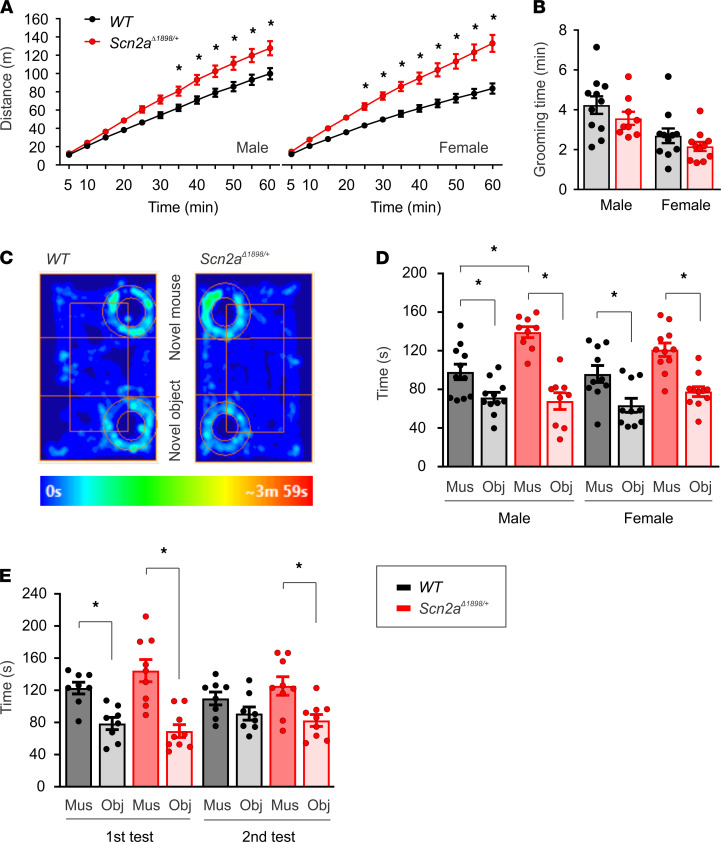
*Scn2a^Δ1898/+^* mice display hyperactivity in a novel environment and show increased social interactions. (**A**) Hyperactivity in *Scn2a*^Δ1898/+^** male and female mice compared with WT littermate controls in an open field (male: WT, *n =* 11 and *Scn2a*^Δ1898/+^**, *n =* 9; female: WT, *n =* 11 and *Scn2a*^Δ1898/+^**, *n =* 11). Asterisks represent 2-way ANOVA followed by Sidak’s multiple-comparison test. Distance × genotype, F_(11, 198)_ = 8.278 for male and F_(11, 220)_ = 16.27 for female, *P <* 0.0001. (**B**) Grooming time. (**C**) Heatmaps from 3 chamber social interaction tests for WT (male, *n =* 11; female, *n =* 10) and *Scn2a*^Δ1898/+^** (male, *n =* 9; female, *n =* 11) mice. (**D**) Time spent with novel mouse (Mus) or novel object (Obj) in the 3-chamber social interaction test. Asterisks represent 2-way ANOVA followed by Tukey’s multiple-comparison test. Mus or Obj × genotype, F_(1, 36)_ = 9.963, *P =* 0.003 for male; F_(1, 38)_ = 0.5996, *P =* 0.445 for female. Mus versus Obj: Tukey’s multiple-comparison test; WT, *P =* 0.04; *Scn2a*^Δ1898/+^**, *P <* 0.0001 for male; WT, *P =* 0.02; *Scn2a*^Δ1898/+^**, *P =* 0.0004 for female. (**E**) Repeated 3 3-chamber social interaction tests in a separate cohort of male WT (*n =* 8) and *Scn2a*^Δ1898/+^** (*n =* 9) mice. Unpaired *t* test was used to evaluate the difference in time spent between Mus and Obj. Both WT (t_(14)_ = 4.174, *P =* 0.0009) and *Scn2a*^Δ1898/+^** (t_(16)_ = 4.721, *P =* 0.0002) mice spent more time with novel Mus over Obj during the first test, but only *Scn2a*^Δ1898/+^** mice displayed a social preference 3 hours later (second test) to the familiar Mus (t_(16)_ = 3.149, *P =* 0.0062).

Having established a Na_V_1.2 loss-of-function defect in *Scn2a*^Δ1898/+^** forebrain neurons, we investigated whether this mutation conferred consequences on behavior. We first assessed general locomotor activity in a novel open-field arena environment. Mutants of both sexes showed no deficits in locomotion, but rather increased locomotion compared with WT mice (Figure 4A). We then assessed core ASD-associated behaviors, repetitive behavior, and social interaction. Using grooming time as a correlate of repetitive behaviors (22), we observed no genotype-specific differences in either males or females (Figure 4B). We then assayed sociability in a 5-minute, 3-chamber social interaction test. Both male and female WT controls and *Scn2a*^Δ1898/+^** mice spent more time with a novel mouse than with a novel object (Figure 4, C and D). However, male *Scn2a*^Δ1898/+^** mice spent more time with the novel mouse compared with their WT controls (Figure 4D). This was also reflected in higher distance traveled by *Scn2a*^Δ1898/+^** compared with WT mice in the zone around the novel mouse (Supplemental Figure 4A), with no difference in distance traveled around the object (Supplemental Figure 4A) and other areas of the 3-chamber apparatus (Supplemental Figure 4, B and C). To further explore this effect, we utilized a modified 3-chamber social interaction protocol to monitor sociability and habituation to a novel mouse, typically seen in WT mice across repeated testing sessions (23, 24). A separate cohort of male *Scn2a*^Δ1898/+^** and WT mice underwent an initial 3-chamber social interaction test (the first test) followed by an additional test (same stranger) 3 hours after the first test. As we observed above (Figure 4, C and D), both WT controls and *Scn2a*^Δ1898/+^** mice showed a social preference during the first test. During the subsequent test, the WT controls showed the expected habituation to the familiar mouse (3 hours afterward; ref. 23), in contrast, the *Scn2a*^Δ1898/+^** mice continued to spend significantly more time with the familiar mouse during the subsequent test (Figure 4E). These data support that *Scn2a*^Δ1898/+^** mice display altered social interaction behavior characterized by sustained time with the social stimulus. Interestingly, human ASD subjects with *SCN2A* mutations were reported to “enjoy physical contact with caregivers” (25). Furthermore, clinical data from the Simons Variation in Individuals Project (VIP) data (v. 3.0) for individuals with *SCN2A* variants whose caregiver completed the Autism Diagnostic Interview-Revised (ADI-R; ref. 26) with a clinician support those observations. In this largest publicly available database of clinical data in patients with *SCN2A* variants (https://www.sfari.org/funded-project/simons-variation-in-individuals-project-simons-vip/), 10 individuals had an *SCN2A* variant and ADI-R data — 7 who were classified as having autism from the Autism Diagnostic Observation Schedule (ADOS; ref. 27), 2 who were classified as nonspectrum, and 1 with missing data. Four of the 10 individuals had a protein truncation variant with a frameshift. The ADI-R includes a question regarding social disinhibition that queries whether the child exhibits behavior that is not appropriately modulated according to the social situation, such as inappropriately friendly (i.e., approaching or touching strangers), or if the child is more socially naive than other children (i.e., unable to understand what to say or do in a particular social situation). Caregiver answers were scored on a 4-point scale. Three children scored “occasional disinhibition”; 5 scored “lack of social inhibition”; and 2 scored “marked social disinhibition.” None reported an absence of disinhibition. Thus, the social interaction abnormality displayed by *Scn2a*^Δ1898/+^** mice correlates well with observations of humans with *SCN2A* variants and with the inappropriate social disinhibition observed in individuals with *SCN2A* mutations.

**Figure 5:**
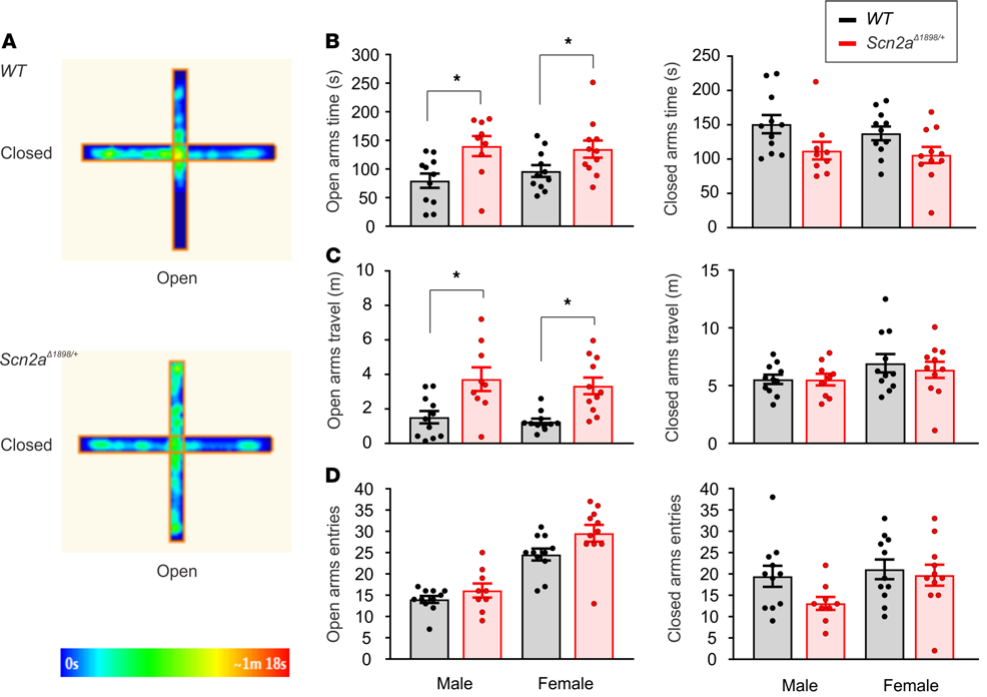
*Scn2a^Δ1898/+^* mice display increased time and traveled distance on the EPM open arms. (**A**) Exemplar heatmaps for WT (male, *n =* 11; female, *n =* 11) and *Scn2a*^Δ1898/+^** (male, *n =* 9; female, *n =* 11) on the EPM. (**B**) Time spent in the open and closed arms. Asterisks represent unpaired *t* test; t_(18)_ = 2.886, *P =* 0.01 for male; t_(20)_ = 2.103, *P =* 0.048 for female. (**C**) Distance traveled in the open and closed arms. Asterisks represent unpaired *t* test; t_(18)_ = 2.998, *P =* 0.008 for male; t_(20)_ = 4.07, *P =* 0.001 for female. (**D**) Number of entries to the open and closed arms.

Inappropriate social contact with strangers, often characterized as over-friendliness, is a feature of Williams syndrome (WS), a neurodevelopmental disorder caused by hemizygous deletion of 7q11.23 (28). A mouse model of WS shows increased social interaction and lack of habituation to a stranger mouse (29), suggestive of the phenotype we observed in the *Scn2a*^Δ1898/+^** mice. While the underlying genetic defect in subjects with WS is distinct from the loss-of-function mutations in *SCN2A*-associated ASD subjects, we considered the possibility that the underlying circuit-level deficits in WS provided guideposts to uncover circuit deficits in ASD subjects with *SCN2A* mutations. A recent review (30) attributed the increased social interaction phenotype in WS subjects to impaired detection of danger, therefore motivating us to test for impaired danger detection in *Scn2a*^Δ1898/+^** mice. We examined performance on an elevated plus maze (EPM) that assesses innate fear and anxiety (31). Both male and female *Scn2a*^Δ1898/+^** mice spent more time, and traveled longer distances, in the open arms than their WT littermate controls (Figure 5, A–C). Neither sex displayed any difference in number of entries to the open arms or closed arms, and there was no difference in time spent and distance traveled in the closed arms (Figure 5, B–D). Therefore, the genotype-specific differences are restricted to the open arms, so the EPM data suggest decreased anxiety-like behavior.

### Neuronal synaptic transmission consequences of a Scn2a severe hypomorphic allele.

**Figure 6:**
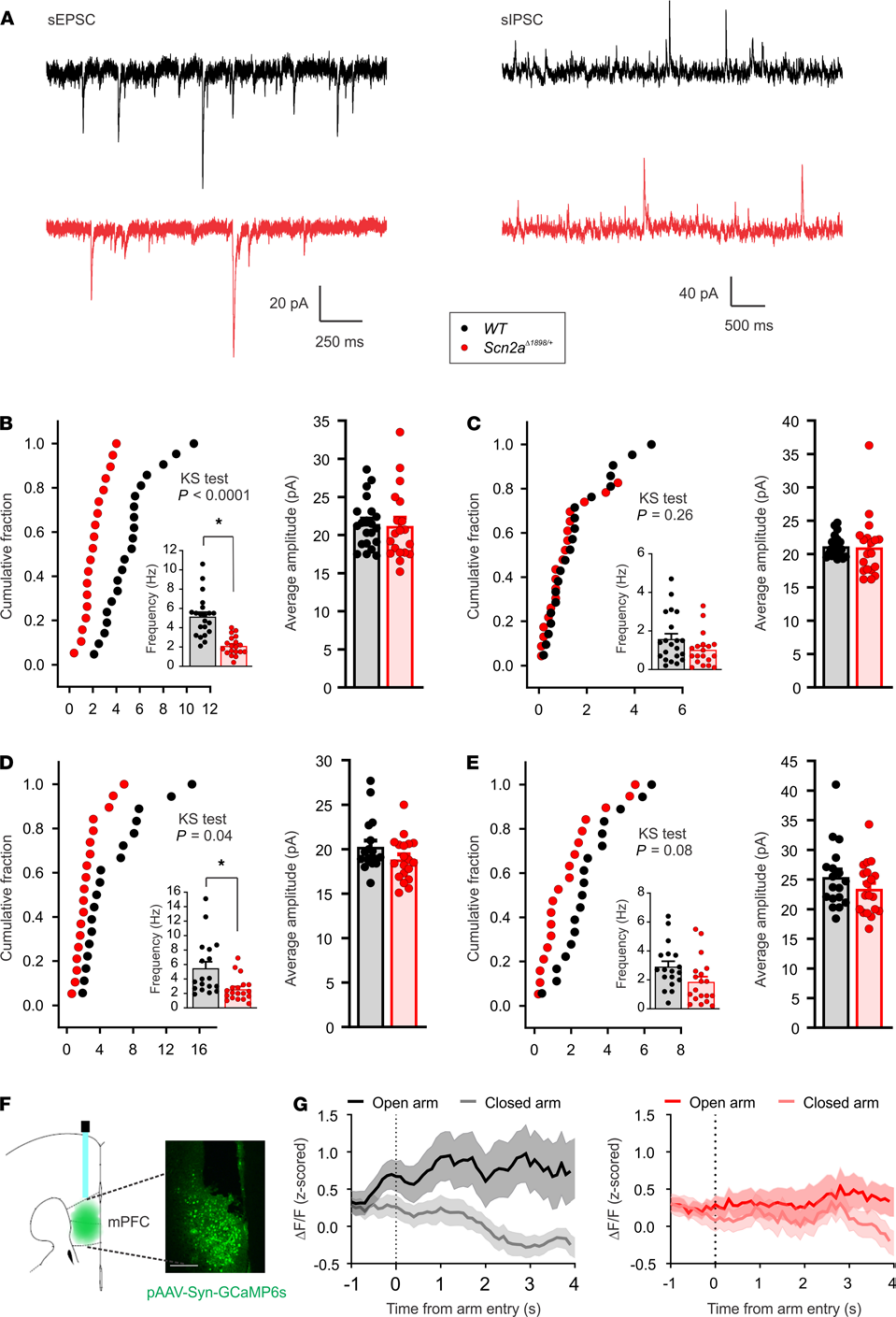
Pyramidal neurons in mPFC and BLA from *Scn2a^Δ1898/+^* mice display altered excitability and synaptic properties. (**A**) Exemplar sEPSCs and sIPSCs recorded in pyramidal neurons from *Scn2a*^Δ1898/+^** and WT mice at holding potential of –50 mV and 0 mV, respectively. WT, black; *Scn2a*^Δ1898/+^**, red. (**B** and **C**) Quantification of frequency and amplitude of sEPSCs and sIPSCs (*Scn2a*^Δ1898/+^**, *n =* 19; WT, *n =* 21) recorded in layer 5/6 pyramidal neurons in mPFC. Frequency of sEPSCs reduced as shown in both cumulative fraction and average (inset, asterisks represent unpaired *t* test, t_(38)_ = 5.691, *P <* 0.0001). (**D** and **E**) Quantification of frequency and amplitude of sEPSCs and sIEPSCs (*Scn2a*^Δ1898/+^**, *n =* 19; WT, *n =* 18) recorded in pyramidal neurons in BLA. Frequency of sEPSCs reduced as shown in both cumulative fraction and average (inset, asterisks represent unpaired *t* test, t_(35)_ = 3.027, *P =* 0.005). The cumulative frequency distributions were analyzed with the Kolmogorov-Smirnov comparison (KS test) in **B**–**E**. (**F**) Microscopic graph showing the GCaMP6s virus expression and fiber placement in mPFC. (**G**) Calcium dynamics in GCaMP6s-expressed neurons with fiber photometry recording during EPM. In contrast to WT mice, *Scn2a*^Δ1898/+^** mice failed to show an increase in fluorescence [Ca^2+^] signal when mice entered the open arms compared with the closed arms.

Both mPFC and amygdala are central hubs integrating distinct functional signals related to higher cognitive and emotional behaviors, including sociability and fear (32–34). Since *Scn2a*^Δ1898/+^** mice display abnormal social interaction and decreased fear-like behavior, we next tested the influence of the decreased neuronal excitability of *Scn2a*^Δ1898/+^** on synaptic properties and on the E/I balance in brain slices with inclusion of mPFC or BLA from adult mice. We measured spontaneous excitatory and inhibitory postsynaptic currents (sEPSCs and sIPSCs, respectively) in pyramidal neurons in layer 5/6 of the mPFC and in the BLA (Supplemental Figure 5, A and B). The frequency of sEPSCs was reduced in *Scn2a*^Δ1898/+^** mice compared with WT littermate controls in both mPFC and BLA pyramidal neurons, suggesting reduced excitatory synaptic input (Figure 6, A, B, and D). The average sIPSC frequency trended toward a decrease but did not reach statistical difference (Figure 6, A, C, and E). On the other hand, sEPSC and sIPSC amplitudes were comparable between WT and *Scn2a*^Δ1898/+^** mice (Figure 6, A–E), suggesting no postsynaptic differences between genotypes. These data, combined with the observed effects on elicited APs and excitability in pyramidal neurons, the observed lack of an effect on Na^+^ currents in inhibitory neurons, and the reported absence of a change in inhibitory neuron excitability (21) suggest that neuronal firing was reduced in *Scn2a*^Δ1898/+^** pyramidal neurons, leading to a reduction in excitatory input to postsynaptic neurons. Overall, this suggests a decrease in excitatory synaptic transmission in *Scn2a*^Δ1898/+^** neurons.

### In vivo neuronal activity consequences of a Scn2a severe hypomorphic allele.

We next examined whether the decreased excitability measured in cultured neurons and the decreased synaptic transmission in slices correlated with decreased activity in vivo and with consequences on behavior. We injected a virus encoding the neuronal activity sensor GCaMP6s into mPFC, since circuits from the amygdala to the mPFC have been implicated in fear (35), and tested calcium dynamics in GCaMP6s-expressing neurons with fiber photometry during EPM (Figure 6F). When traveling into an open arm on the EPM, WT mice showed an increase in neuronal activity starting within 1 second of open arm entry, but they showed no increase when entering a closed arm. In contrast, *Scn2a*^Δ1898/+^** mice displayed no increase in activity when entering either the open or closed arm (Figure 6G). This result echoes the decreased neuronal activity observed in the *Scn2a*^Δ1898/+^** mice in the cellular and slice recordings, and it provides a correlation between abnormal neuronal activity and abnormal behavior in *Scn2a*^Δ1898/+^** mice.

## Discussion

Although next-generation sequencing shows that de novo loss-of-function protein truncation variants in *SCN2A*, often associated with a frameshift, are among the most common genetic associations with ASD (7, 8), the mechanisms leading to ASD-associated endophenotypes have not been definitively determined. Here, we demonstrated that an *Scn2a* protein truncation variant that appears to be rapidly degraded reduced the number of functional channels and the amount of Na_V_ current in excitatory neurons in which *Scn2a* is predominantly expressed. Consistent with reduced Na_V_1.2, we found that *Scn2a*^Δ1898/+^** pyramidal neurons were less excitable. Our observations in mouse neurons, slices, and in vivo echo a report in which knockout of *SCN2A* in neurons derived from induced pluripotent stem cells reduced extracellular spontaneous network activity in glutamatergic neurons (36).

Moreover, our analyses reveal correlations between the cellular abnormalities and changes in behavior in *Scn2a*^Δ1898/+^** mice that, to date, have been elusive when considering the various reported studies of *Scn2a^+/–^* models. Those studies (11, 12, 14, 15) have shown marked discrepancies in the autistic-like and other ASD comorbid behavior phenotypes. For instance, some studies reported increased social interaction (11, 14), while others reported a mild decrease (12, 15); some studies reported increased grooming in a novel environment (14, 15), while another failed to detect abnormal grooming (11). The reasons for the discrepant results, despite using identical or nearly identical models, is not clear. Thus, the utility of comparing a different *Scn2a* haploinsufficient model provides an opportunity to define which endophenotypes most likely result from reduced Na_V_1.2 expression.

While we cannot completely rule out a contribution of the additional 26 amino acids generated by the frameshift in the *Scn2a*^Δ1898/+^** mice, our data suggest that the additional 26 amino acids have no discernible effect on Na_V_1.2 channel function; a Na_V_1.2 protein with a stop codon inserted at T1898 displayed identical channel properties, as shown in Figure 1. Moreover, the truncated and frameshifted Na_V_1.2 behaves in vivo like a severe hypomorphic allele, as suggested by several lines of evidence. First, the complete absence of surviving *Scn2a*^Δ*1898/*Δ1898^** pups is consistent with the perinatal lethality of *Scn2a^–/–^* mice (20). Secondly, our biochemical and electrophysiological analyses suggest little, if any, functional protein from the mutant allele (Figure 2 and Figure 3). Therefore, by demonstrating functionally equivalent biophysical and biochemical consequences of the truncation and the frameshift variant in a heterologous expression system, our data show that the reduced Na^+^ channel function recorded in neurons isolated from *Scn2a*^Δ1898/+^** mice result from the eliminated Na_V_1.2 protein rather than the introduction of the additional 26 amino acids. Thus, the hypomorphic *Scn2a*^Δ1898/+^** model is representative of ASD-associated mutations in *SCN2A*, such as D82G or T1420M (10).

With that background, the *Scn2a*^Δ1898/+^** mice displayed an increase in social interaction (consistent with refs. 11, 14) and an increase in locomotor activity, along with reduced anxiety-like behavior (consistent with refs. 12, 14, 15). We also observed no change in grooming in *Scn2a*^Δ1898/+^** mice, consistent with the previous report by Shin, et al. (11). Thus, the overlaps with our distinct model suggests validation for those behavior abnormalities as indeed associated with *Scn2a* haploinsufficiency. Additionally, the hypersocial interaction phenotype, reported by several groups using a 3-chamber social interaction test for various mouse models (14, 29, 37–40) and specifically in the *Scn2a^+/–^* model (11, 14), appears to be consistent with the inappropriate social approach behavior (i.e., inappropriate social disinhibition) reported in individuals with *SCN2A* variants seen in our analysis of the Simons VIP database. Furthermore, our fiber photometry data suggest a direct link from cellular electrophysiology and a specific behavior in the mouse model.

Having established social disinhibition and decreased anxiety (on the EPM) as phenotypes associated with decreased *Scn2a* expression, analysis of circuit deficits in WS may serve as a guide for future studies to investigate the consequences of *Scn2a* haploinsufficiency. Functional MRI in individuals with WS showed reduced functional connectivity between the amygdala and the medial prefrontal and orbitofrontal cortex, which was correlated with social disinhibition (41). Recent studies demonstrated that the mPFC makes excitatory projections to principal neurons in the BLA (42, 43). We therefore hypothesize that the loss-of-function defects observed in Na^+^ currents within pyramidal neurons from *Scn2a*^Δ1898/+^** mice, and the consequent reduced excitatory input, impair the excitatory connections from the medial prefrontal to the BLA. This, in turn, would reduce amygdala output. The abnormal behavior was correlated to a reduced neuronal activity in mPFC in fiber photometry recording in *Scn2a*^Δ1898/+^** mice. Consistent with this hypothesized reduced amygdala output, the *Scn2a*^Δ1898/+^** mice demonstrated an impairment in danger detection, as observed on the EPM. The similarities among the social disinhibition in humans with *SCN2A* variants, the reported enjoyment of interactions with caregivers for humans with *SCN2A* variants, and the observed social phenotypic abnormality in the *Scn2a*^Δ1898/+^** mouse model suggest a testable cellular-to-endophenotype connection for future studies. Future circuit level analyses will allow us to test this hypothesis in detail, but the data here suggest that a neocortical E/I imbalance, whether an increased or decreased ratio, is a substrate for ASD-associated endophenotypes. It is possible that the heterogeneous changes in E/I imbalance (higher or lower) and the consequent alteration in brain network in the temporal and spatial scales could be associated with different manifestations of behavior, which is reflected by the clinical heterogeneity of autistic patients.

While decreased excitability in the principal neurons expressing *Scn2a* with decreased frequency of sEPSCs provides a rationale for that proposed deficit in the prefrontal cortex to amygdala circuit, additional factors may also contribute. We did not observe a significant difference in frequency of sIPSCs between WT and *Scn2a*^Δ1898/+^** mice, nor a difference in Na^+^ currents within inhibitory neurons, although the average frequency of sIPSCs showed a trend to decrease. The subtle reduction in sIPSCs could represent a homeostatic response aimed at maintaining the proper E/I balance through local synaptic adaptions and network-wide adjustments (44), but this change in inhibitory input could not restore the overall deficit in excitatory activity of pyramidal neurons.

We found that the difference in the AP amplitude between *Scn2a*^Δ1898/+^** and WT littermate control neurons was no longer observed with a stimulus that elicited a maximal AP amplitude, suggesting that the reduced AP amplitude with a smaller stimulus derived mainly from the reduced Na^+^ current amplitude. However, the higher intensity stimulus did not correct the AP threshold, suggesting that the observed differences in AP between WT and *Scn2a*^Δ1898/+^** (Figure 3E and Table 2) also reflected compensation by other non-Na^+^ channel ionic currents. Likely, other homeostatic mechanisms, at the cellular and circuit levels, are activated even though those mechanisms appear insufficient to rescue the hypersocial behavior or the decreased anxiety-like behavior and/or impairment of danger detection as indicated by the EPM.

Excitatory neurons within the cortex express both Na_V_1.2 and Na_V_1.6, yet mutations in *SCN8A* that encodes Na_V_1.6 have not been associated with ASD. That expression of Na_V_1.6 is not prominent until later developmental stages, when critical neural circuits are already established; this provides at least one likely reason why *SCN8A* mutations have not been associated with ASD. Consistent with a later role in development for Na_V_1.6, we did not observe a compensatory increase in *Scn8a* mRNA, or any other CNS Na^+^ channel, in the P6 and adult *Scn2a*^Δ1898/+^** mice. This absent compensatory response to a reduction in Na_V_1.2 protein and Na_V_1.2-dependent Na^+^ current in the *Scn2a*^Δ1898/+^** mice may offer an additional explanation for why loss-of-function *SCN2A* mutations have been identified as one of the most common genetic associations with ASD.

In summary, the cellular electrophysiology data and the behavior data displayed by the *Scn2a*^Δ1898/+^** mice, and the resulting protein truncation variant Na_V_1.2^Δ1898^, provide valuable tools to investigate the molecular mechanisms by which *SCN2A* mutations cause abnormal social behaviors and offer opportunities to explore therapeutic options for one of the most commonly affected ASD-associated loci.

## Methods

### Frameshift generation.

The *Scn2a*^Δ1898/+^** mouse line with a frameshift (p.T1898NfsX27) in 1 allele of *Scn2a* (Figure 1A) was created by CRISPR/Cas9 by the Transgenic Mouse Shared Resource at Duke University School of Medicine (Durham, North Carolina, USA) during an attempt to create a *Scn2a^R1903C^* knock-in mutation. The guide sequence (5′-GATAGCGTCTGTAAGCTCGCTGG-3′) was identified using the online tool at http://crispr.mit.edu/ The guide and repair oligonucleotides (5′-GCTCTTCGAATCCAGATGGAAGAGCGGTTCATGGCTTCCAATCCCTCCAAGGTCTCTTATGAGCCCATTACCACCACTCTGAAG**T**GCAAACAAGAGGAGGTGTCTGCTATTGTCAT**A**CAGCGAGCTTACAGACGCTATCTTCTGAAACAGAAAGTTAAGAAGGTTTCGTCTATATATAAAAAAGACAAGGGTAAAGAAGA-3′; the underlined “T” introduces a C>T point mutation, and the underlined “A” disrupts the PAM sequence and creates a silent mutation within the coding region) were synthesized at Integrated DNA Technologies Inc. and subcloned into pX330 using standard cloning procedures, and they were produced from recombinant pX330 plasmid using MEGAshortscript (Ambion) following the manufacturer’s protocol. sgRNAs were tested in vitro using Guide-it sgRNA Screening System (Clontech Laboratories) following the manufacturer’s protocol. The reagents were injected into B6SJLF1/J oocytes to obtain pups that were sequenced by sequencing genomic DNA by PCR. A line with the off-target p.T1898NfsX27 (Supplemental Figure 1) in 1 allele was selected for further study.

### Heterologous expression system analyses of voltage-gated currents and biotinylation.

QuikChange Site-Directed Mutagenesis kit (Agilent Technologies) was used to generate p.T1897NfsX27 (Na_V_1.2^Δ1897^, equivalent to mouse p.T1898NfsX27) in a human *SCN2A* cDNA subcloned into pCI-Neo. The truncation mutant (Na_V_1.2^1897–STOP^) was also generated with QuikChange, by inserting a stop codon after T1897 in the *SCN2A* cDNA.

### Biotinylation analysis.

HEK293 cells were cultured in DMEM with 10% FBS in a 37°C incubator with 5% CO_2_ and were grown in 100 mm culture dishes. Plasmids encoding WT Na_V_1.2 (Na_V_1.2^WT^), Na_V_1.2^Δ1897^, Na_V_1.2^1897–STOP^, or empty vector (8 μg) were cotransfected with *Scn1b* (4 μg) using Lipofectamine 2000 (Thermo Fisher Scientific). Forty-eight hours after transfection, cultured HEK293 cells were washed with ice cold PBS and incubated in 1 mg/mL sulfo-NHS-SS-Biotin (Thermo Fisher Scientific) in PBS for 30 minutes at 4°C. Cells were washed twice with 100 mM glycine to quench the reaction and then were lysed in lysis buffer (NaCl 150 mM, Tris 50 mM, Triton 1%, and Roche protease inhibitor 1 tablet/7.5 mL, pH 7.4). After rocking for 30 minutes at 4°C, lysates were passed through 18 gauge and then 25 gauge needles for 25 passes each; they were then centrifuged at 17,000*g* for 15 minutes at 4°C. Supernatants were collected and protein concentration was quantified using a BCA Protein Assay Kit (Thermo Fisher Scientific). Supernatants were incubated and rocked with NeutrAvidin agarose resin (Thermo Fisher Scientific) overnight at 4°C. The following day, the beads were washed 3 times with lysis buffer, resuspended in LDS sample buffer (Thermo Fisher Scientific) containing 50 mM DTT, and heated to 95°C for 3 minutes. About 20 g of protein were dissolved with LDS sample buffer and separated on Novex WedgeWell 8 to 16% Tris–glycine gels (Thermo Fisher Scientific) and transferred to PVDF blotting membrane (GE Healthcare Life Sciences). The membrane was immunoblotted with an anti–pan sodium channel antibody (1:1000; MilliporeSigma, S8809), anti–transferrin receptor (1:1000; Thermo Fisher Scientific, 13-6800) and anti–β-actin (1:5000; MilliporeSigma, A1978) antibodies and detected by chemiluminescence. Images were captured using Kodak Image Station 4000 R and quantified using ImageJ (NIH).

### Immunoblotting of brain cortex.

About 80 mg neonatal (P6) and adult (~5-month-old) brain cortices were homogenized with glass homogenizer in RIPA Buffer (Thermo Fisher Scientific) containing NaCl 150 mM, Tris-HCl 25 mM, NP-40 1%, sodium deoxycholate 1%, SDS 0.1%, ThermoHalt protease inhibitor, and Halt phosphatase inhibitor, pH 7.6 (Thermo Fisher Scientific). After rocking for 2 hours at 4°C, lysates were passed through 18 gauge and then 25 gauge needles for 25 passes each; they were then centrifuged at 17,000*g* for 15 minutes at 4°C. Supernatants were collected, and protein concentration was quantified using a BCA Protein Assay Kit (Thermo Fisher Scientific). Protein (30 µg) was separated on Novex WedgeWell 8 to 16% Tris–glycine gels (Thermo Fisher Scientific) and transferred to PVDF blotting membrane (GE Healthcare Life Sciences). The membrane was immunoblotted with anti–pan sodium channel antibody (1:1000; MilliporeSigma, S8809), anti–Na_V_1.2 sodium channel (1:200; NeuroMab, 75-024; Alomone Labs, ASC-002), and anti–β-actin (1:5000; MilliporeSigma, A1978) antibodies. The blots were visualized by chemiluminescence, and images were captured using ChemiDoc Touch Imaging System (Bio-Rad) and quantified using ImageJ (NIH).

### IHC of brain cortex.

Brains were rapidly removed from newborn pups and immersed in 0.5% paraformaldehyde in 0.1M PBS for 2 hours at 4°C followed by an overnight immersion in 30% sucrose in PBS. Then, brains were embedded in OCT compound and stored at –80°C overnight. Brain tissue was sectioned to 10 μm slices using a cryostat, and slices were stored overnight in –20°C. Brain sections were thawed for 5 minutes in room temperature, rinsed with 0.1M PBS, and incubated with primary antibodies: a custom rabbit Na_V_1.2 (1:500) designed by YenZym LLC and mouse ankyrin G (1:200; NeuroMab, N106/36) in PBS with 5% bovine albumin serum and 0.3% Triton X-100 overnight at 4°C. Subsequently, after slices were rinsed with 0.1M PBS 3 times each for 5 minutes, the slices were incubated with secondary antibodies Alexa Fluor 488 anti-rabbit and Alexa Fluor 647 anti–mouse IgG (1:500) at room temperature (25°C) for 1 hour. Slides were then washed with 0.1M PBS for 3 times each for 5 minutes and were mounted using mounting media (Vector Laboratories) and sealed with a glass cover slip. Slides were kept at 4°C for 2 hours. Stained brain slices were imaged using a confocal microscope (Zeiss LSM 880), and *Z* stacks were obtained with 0.5 μm per slice. Images were merged and analyzed using ImageJ software.

### qPCR.

Total mRNA was purified from neonatal (3–6 days) and adult (~5-month-old) brain cortices using RNAeasy Plus Mini kit (Qiagen), and it was reverse transcribed to single-stranded cDNA library using iScript cDNA Synthesis Kit (Bio-Rad). Primers for qPCR were 5′-CACTCATTATTCAGCATGTTAATCATGTGC-3′ (forward) and 5′-CGATGGTCTTCAGGCCTGGAATG-3′ (reverse) for *Scn1a*; 5′-CCAGACTGGACAAAGAATGTGGAGTATAC-3′ (forward) and 5′-CGATGGTCTTCAGGCCTGGAAT-3′ (reverse) for *Scn2a*; 5′-CCTGACTGGACGAAGAATGTAGAGTACAC-3′ (forward) and 5′-CGATGGTCTTTAAACCTGGAATGACTG-3′ (reverse) for *Scn3a*; 5′-CATTCAGTCTTCAGCATGATCATCATGTG-3′ (forward) and 5′-CGATTGTCTTCAGGCCTGGGAT-3′ (reverse) for *Scn8a*; and 5′-CCTGGAGAAACCTGCCAAGTATGATG-3′ (forward) and 5′-CTGTAGCCGTATTCATTGTCATACCAGG-3′ (reverse) for GAPDH. qPCR (a total 40 cycles) was performed using the QuantStudio 3 (Applied Biosystems). The relative amount of target message in each reaction was determined from the detection threshold cycle number (Ct), which was normalized to the Ct for GAPDH obtained simultaneously.

### Forebrain cortex neuron cultures.

Forebrain cortices from 1- to 3-day newborn WT and p.T1898NfsX27 (*Scn2a*^Δ1898/+^**) mice were dissociated through enzymatic treatment with 0.25% trypsin and subsequent trituration. The cells were plated on glass coverslips previously coated with poly-D-lysine and laminin in 12-well cell culture plate in the density of 170,000/mL. The cortical cells were grown in neurobasal A medium (Thermo Fisher Scientific) supplemented with B-27 2%, glutamine 2 mM, heat-inactivated FBS 10%, and penicillin/streptomycin 1% in 5% CO_2_ incubator at 37°C overnight; then, this medium was replaced by one containing B-27 2%, glutamine 0.5 mM, heat-inactivated FBS 1%, uridine 70 μM, and 5-fluorodeoxyuridine 25 μM. Cultured neurons were used for electrophysiology 5 days in vitro (DIV) after plating, and recordings were performed on either pyramidal or nonpyramidal shaped neurons selected based on the shape of their cell body and dendritic pattern (Supplemental Figure 2, A and B).

### Acute slice preparation.

Coronal brain slices were prepared from male and female *Scn2a*^Δ1898/+^** mice and their respective littermates (8–10 weeks old). Animals were anesthetized with 1.25% Avertin (250 mg/kg; i.p.) and then transcardially perfused with a cold sucrose-based solution. This solution contained (in mM): sucrose 220, KCl 2.5, MgSO_4_ 12, CaCl_2_ 0.5, NaH_2_PO_4_ 1.25, NaHCO_3_ 26, glucose 10, HEPES 10, and sodium pyruvate 3 (pH 7.4). After decapitation, the brain was transferred quickly into the above-mentioned ice-cold sucrose based cutting solution bubbled with 95% O_2_ and 5% CO_2_. Coronal brain slices (300 μm) including mPFC or BLA were prepared using a Leica VT1200S vibratome (Leica), and they were incubated in a BSK-2 brain slice keeper (Automate Scientific) containing oxygenated artificial cerebrospinal fluid (aCSF) at 35°C for 40 minutes. Afterward, the slices were maintained at room temperature at least 30 minutes before use.

### Electrophysiology.

Whole-cell sodium (Na^+^) currents from HEK293 cells and cultured neurons, and sEPSC and sIPSC from acute brain slices, were recorded in the voltage patch-clamp configuration, and APs from cultured neurons were recorded in the current-clamp configuration with an Axopatch 200B amplifier (Molecular Devices) and sampled at 10 kHz and filtered at 2 kHz. Data were analyzed with Axon Clampfit (Molecular Devices) or Mini Analysis (Synaptosoft Inc.).

For Na^+^ currents recording in HEK cells, the pipette internal solution contained the following (in mM): CsCl 16, CsF 110, NaCl 10, CaCl_2_ 0.5, MgCl_2_ 1, EGTA 10, HEPES 10, Na_2_-APT 2 (pH 7.3), with CsOH. The external solution contained (in mM): NaCl 120, KCl 5.4, CaCl_2_ 1.8, MgCl_2_ 1, HEPES 10, glucose 10, tetraethylammonium chloride (TEA-Cl) 20 (pH 7.4), with NaOH. For Na^+^ currents recording in cultured neurons, the pipette internal solution contained (in mM): CsCl 50, CsF 35, L-aspartic acid 55, NaCl 10, EGTA 5, MgCl_2_ 1, Mg-ATP 4, Na_2_-GTP 0.4, and HEPES 10 (pH 7.3), with CsOH. External solution contained (in mM): NaCl 100, KCl 5, HEPES 20, CaCl_2_ 2, MgCl_2_ 2, glucose 30, TEA-Cl 20, 4-aminopyridine 2, CdCl_2_ 0.5, APV 0.05, DNQX 0.02, and bicuculline 0.02 (pH 7.4), with NaOH. For AP initiation in cultured neurons, the pipette internal solution contained (in mM): potassium gluconate 130, KCl 10, MgCl_2_ 5, EGTA 0.6, HEPES 5, CaCl_2_ 0.06, phosphocreatine disodium 10, Mg-ATP 2, Na_2_-GTP 0.2, and creatine phosphokinase 50 U/mL (pH 7.2), adjusted with KOH. The external solution contained (in mM): NaCl 119, KCl 5, HEPES 20, CaCl_2_ 2, MgCl_2_ 2, glucose 30, APV 0.05, DNQX 0.02, and bicuculline 0.02 (pH 7.3), adjusted with NaOH. For sEPSCs and sIEPSCs recording from brain slices, the pipette internal contained (in mM): potassium gluconate 125, KCl 10, MgCl_2_ 5, EGTA 0.6, HEPES 5, CaCl_2_ 0.06, phosphocreatine disodium 10, Mg-ATP 2, Na_2_-GTP 0.2, creatine phosphokinase 50 U/mL, and lidocanine N-ethyl bromide 5 (pH 7.2), adjusted with KOH. The aCSF contained (in mM); NaCl 126, KCl 2.5, NaH2PO_4_ 1.25, NaHCO_3_ 26, CaCl_2_ 2, MgCl_2_ 2, and glucose 10. Equilibrium voltage across sEPSCs and sIPSCs recording solutions for Cl^–^ (E_Cl_) and cation ions (E_cation_) were –51 mV and 0 mV, respectively.

For recording Na^+^ currents in HEK293 cells, cells were cotransfected with Na_V_1.2^WT^*,* Na_V_1.2^Δ1897^, or Na_V_1.2 Na_V_1.2^1897–STOP^ plasmid (2 μg) with *Scn1b* (2 μg) and EGFP (0.2 μg) and grown in 60 mm culture dishes for 48 hours before recording. Na^+^ currents were elicited with a 50 ms depolarization step from –100 mV with 5 mV increments at a holding potential of –120 mV. Steady state inactivation was tested by a 2-pulse protocol with the first pulse of 500 ms from –140 mV to –20 mV at 5 mV increments, followed by a second pulse fixed at –20 mV. Gating activation curves were obtained using a Boltzmann function: G/G_max_ = (1 + exp[–(V – V_1/2_)/*k*])^–1^, where G/G_max_ is the conductance normalized to its maximal value, V is the membrane potential, V_1/2_ is the membrane voltage at which the current amplitude is half-maximal, and *k* is the slope factor. For steady state inactivation, Na^+^ currents induced by the second pulse were normalized to the maximal current and plotted as the function of the voltages elicited by the first pulse, which was also fitted with Boltzmann function: I/I_max_ = (1 + exp[(V – V_1/2_)/*k*])^–1^, where I/I_max_ is the normalized value. Neuronal Na^+^ currents were recorded in pyramidal and nonpyramidal shaped neurons in 5–7 DIV culture at the holding potential of –100 mV. AP was elicited with 5 ms or 500 ms depolarization current in pyramidal shaped neurons from 6–9 DIV cultures, and the resting membrane potential was held around –60 mV with current injection.

Brain slices were placed in a recording chamber on the stage of an upright, infrared-differential interference contrast microscope (BX51WI, Olympus Optical) equipped with an ORCA-Flash2.8 C11440 Digital CMOS Camera (Hamamatsu Photonics), and they were continuously superfused at a rate of 2 mL/min with aCSF bubbled with 95% O_2_ and 5% CO_2_ at 35°C ± 0.04°C. mPFC layer 5/6 or BLA pyramidal neurons were visualized with a 40× water-immersion objective. sEPSCs were recorded at –50 mV (near E_Cl_), and sIPSCs were obtained at 0 mV (near E_cation_).

Recording pipettes were pulled from borosilicate glass with Sutter P-97 Micropipette Puller (Sutter Instrument Co). Pipette resistance ranged from 1.9 to 2.9 MΩ and 2.9 to 4 MΩ; series resistance was 6.4 ± 0.3 MΩ and 8.6 ± 0.5 M, compensated by 80% and 60%–70% for Na^+^ current recording in HEK cells and neurons, respectively. The pipette resistance ranged from 2.7 to 6.0 MΩ and series resistance was 11.1 ± 0.3 MΩ without compensation for the recordings of postsynaptic currents in brain slices. In current patch-clamp experiments, input resistance was 222 ± 12.7 MΩ for WT and 218 ± 20.2 MΩ for *Scn2a*^Δ1898/+^**. Junction potential was measured immediately after recording by quickly detaching the pipette from the recorded cell. The measured junction potential was 2.2 ± 0.3 mV for WT and 1.4 ± 0.2 mV for *Scn2a*^Δ1898/+^**, and data were not corrected.

### Behavior analyses.

Male and female heterozygous mice and their respective littermates (3–5 months old) were randomly assigned into experimental groups to perform locomotion, EPM, 3-chamber social interaction, and grooming behavioral tests. Specific protocols for these behavior tests were previously described (45). Male and female *Scn2a*^Δ1898/+^** mice were compared with their respective male and female WT littermate controls, and the investigator analyzing behaviors was blinded to the genotype.

### Locomotor activity measurement.

The locomotor activity of WT and *Scn2a*^Δ1898/+^** mice was measured in a 27.3 × 27.3 cm open-field locomotor activity chamber using open-field activity software (MED Associates). Distance traveled during the 1-hour test period was evaluated.

### Grooming test.

Grooming assay was performed in a dimly lit room (~150 lux). Mice were allowed to habituate to the testing room for at least 30 minutes prior to the initiation of the assay. Each mouse was placed into a housing cage with low bedding, and its behaviors were recorded for 30 minutes. A new clean housing cage was used for each mouse. Grooming behavior was autoscored using pretrained autoscoring software validated previously (46).

### Three-chamber social interaction test.

The social test apparatus consisted of a box divided into 3 chambers of equal size 20 cm (length) × 40.5 cm (width) × 22 cm (height). A testing mouse was introduced to the middle chamber and left to habituate for 5 minutes, followed by another 5-minutes, 3-chamber habituation by removing the retractable walls. After that, the mouse was briefly confined back to the middle chamber; then, an unfamiliar mouse (stranger) was introduced into a wire cage in one of the side chambers, and an object was introduced into a wire cage on the opposite side chamber. The testing mouse was allowed to freely explore all 3 chambers for 5 minutes after removing the retractable walls. All activities of the mouse in the chambers were recorded using AnyMaze software (Stoelting Co). Time spent in each chamber and time spent in the contact zone (1.5” radial diameter around the cup surrounding the wire cage containing the stranger mouse or object) were collected to evaluate sociability. The location of the stranger mouse and object were alternated between the 2 side chambers across the mice tested to avoid a side preference bias. Stranger mice (C57BL/6 male, 3 months old) were purchased from the Jackson Laboratory. Prior to behavioral testing, mice were habituated to the behavioral suite on each testing day.

### EPM test.

The EPM test was performed in an apparatus with 2 open arms and 2 closed arms. Two open arms (25 × 5 × 0.5 cm) across each other were perpendicular to 2 closed arms (25 × 5 × 16 cm) with a center platform (5 × 5 × 0.5 cm). The maze was elevated 40 cm above the floor. Experiments started by placing a mouse on the central platform facing an open arm. During the 5 minutes if free exploration, the number of entries into each arm, the time spent, and distance traveled in the arm were recorded using AnyMaze software (Stoelting Co.).

### Stereotaxic viral delivery and fiber photometry.

As previously described (47), the cohort of mice used for the EPM test in conjunction with fiber photometry recording were anesthetized with isoflurane, and 0.2 μL pAAV1-Syn-GCaMP6s-WPRE-SV40 (Addgene) was unilaterally injected into mPFC (AP: +2.00 mm, ML: −0.25 mm, DV: −2.25 mm). During the same surgery, a 400 μm diameter optical fiber (Doric) was implanted above the injection site in the mPFC (AP: +2.00 mm, ML: −0.25 mm, DV: −2.15 mm) and was secured with Metabond. Animals were tested 3 weeks after surgery.

Fiber photometry was performed to measure in vivo calcium dynamics during EPM. Mice were habituated to the patch cord 1 min in their home cage prior to behavioral testing. During the 5-minute test, a 470 nm LED excitation light (M470F3, Thorlabs) delivered at 521 Hz was passed through a filter (FF02-472/30, Semrock) and reflected by a dichroic glass (FF495-Di03, Semrock); it was coupled to the 0.48 NA, 400 μm core optical fiber patch cord (Doric) in order to excite GCAMP6s within neurons. Emitted fluorescence signals traveled back through the patch cord, passed through a dichroic filter (FF01-535/50, Semrock), and were captured by a photodetector (Model 2151, Newport). The modulated signal was demodulated and low-pass filtered using a corner frequency of 15 Hz through a RP2.1 real-time processor (Tucker Davis Technologies). A TTL pulse was sent to the processor at the start of each behavioral trial to allow the alignment of calcium signal recording to mouse behavior. Following behavioral testing, fluorescence microscopy was used to confirm GCAMP6s expression and optical fiber placement. Mice with improper placements were eliminated from the analyses.

Data were analyzed using a custom MATLAB script. Raw fluorescence signals were first detrended to account for any photobleaching by fitting a third-degree polynomial in 15-second time windows and subtracting this polynomial from the raw signal trace to calculate the change in florescence (ΔF/F). Next, the fluorescence signal was time locked to behavior as defined by entering the open or closed arm. Data were converted to a *Z* score to account for variability in the dynamic range of the signal across animals and trials, and neural activity was quantified as the mean *Z* score. To quantify the fluorescence level prior to arm entries during EPM, the mean *Z* score was calculated over the 1 second prior to every arm entry as determined from behavioral data.

### Simons VIP analysis.

The Simons VIP v.3.0 data were queried for individuals who had an *SCN2A* variant and also had completed in-person clinical phenotyping. Autism diagnoses were confirmed with the ADOS (27). Caregivers completed the ADI-R, a standardized interview that is administered by a trained clinician with research reliability in administration and scoring (26). The ADI-R asks about current and past behaviors. Analyses focused on the social disinhibition item, and caregiver responses were coded into 4 categories (normal social disinhibition, occasional social disinhibition, definite social disinhibition, and marked social disinhibition). Data were analyzed from current behavior only.

### Statistics.

Numerical averages are presented as mean ± SEM. Unless otherwise stated, statistical significance was calculated using the 2-tailed, unpaired *t* tests; 1-way ANOVA; or 2-way ANOVA followed by a multiple-comparison test, based on the specific data set.

### Study approval.

The study was approved by Weill Cornell Medical College Animal Care and Welfare Committee (protocol no. 2016-0042). Animals were handled according to *Guide for the Care and Use of Laboratory Animals* (National Academies Press, 2011).

## Author contributions

HGW, FSL, AMR, and GSP designed research studies; HGW, CCB, AL, and YB conducted experiments; HGW, CCB, AL, and YB acquired data; HGW, CCB, AL, JH, RMJ, YB, AMR, and GSP analyzed data; and HGW, AMR, and GSP wrote the manuscript.

## Supplementary Material

Supplemental data

## Figures and Tables

**Table 1:**
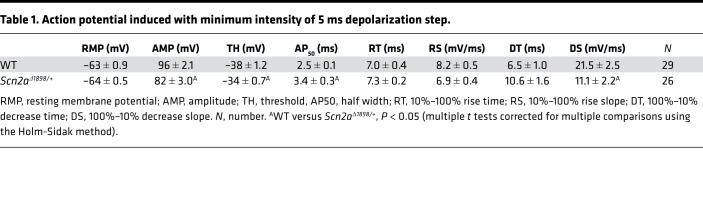
Action potential induced with minimum intensity of 5 ms depolarization step.

**Table 2:**
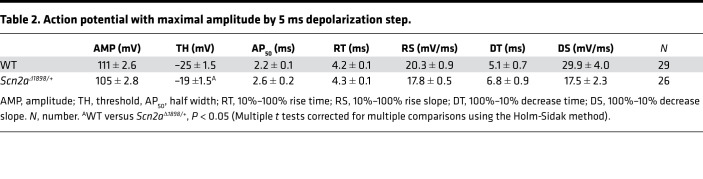
Action potential with maximal amplitude by 5 ms depolarization step.

## Version 1 06/22/2021

In-Press Preview

## Version 2 08/09/2021

Electronic publication
