# Supplementary material for: Scn2a severe hypomorphic mutation decreases excitatory synaptic input and causes autism-associated behaviors
Source: JCI Insight. 2021 Aug 9;6(15):e150698. doi: 10.1172/jci.insight.150698 (PMC8410058; doi:10.1172/jci.insight.150698)

# SUPPLEMENTAL FIGURES FOR:

# *Scn2a* loss-of-function mutation decreases excitatory synaptic input and causes autism-associated behaviors

**Running title: *Scn2a* mouse model shows autism-associated behaviors**

Hong-Gang Wang[1], Charlotte C. Bavley[2,3], Anfei Li[2], Rebecca M. Jones[4,5,6], Jonathan Hackett[3], Yared Bayleyen[1], Francis S. Lee[2,5,6], Anjali M. Rajadhyaksha[2,3,5], Geoffrey S. Pitt[1,5]

**Supplementary Figure 1**

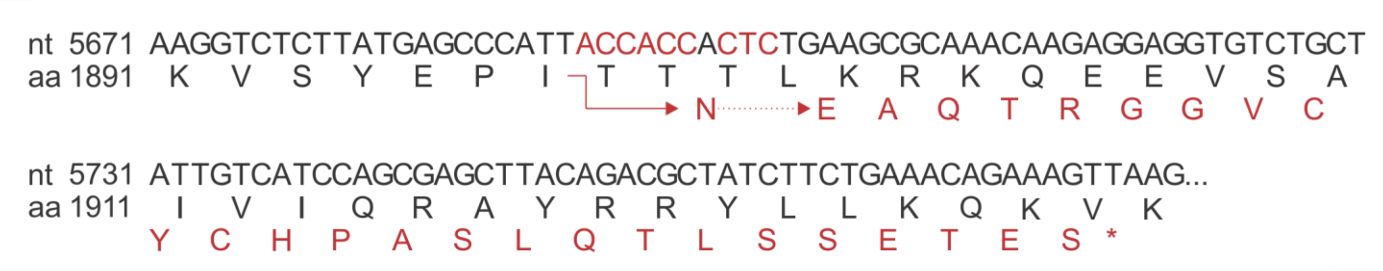


**Supp. Figure 1.** Frameshift and consequence amino acid sequence for $Na_V1.2$ in *Scn2a*$^{\Delta1898/+}$ mice. The red nucleotides were deleted in the indel (see chromatogram in **Figure 1**), resulting in the alternative amino acid sequence (red) and premature STOP codon.

**Supplementary Figure 2**

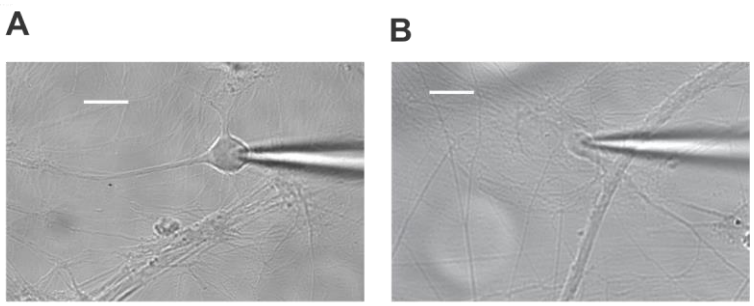


**Supp. Figure 2.** Pyramidal (*A*) and non-pyramidal (*B*) shape neurons in neuronal culture.  Scale bar, 20 µm.

**Supplementary Figure 3**

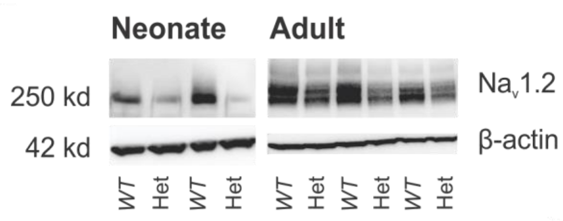


**Supp. Figure 3.** Cortices from *Scn2a*$^{\Delta 1898/+}$ mice have less Na$_V$1.2 voltage-gated $Na^+$ channels. Immunoblot for Nav1.2 $Na^+$ channel (anti-Nav1.2, recognizes an epitope in the I-II intracellular loop) in brain cortex lysates from P6 or adult (~5 month) *WT* or *Scn2a*$^{\Delta 1898/+}$ (Het) mice. Molecular weights are shown on the left. For both blots, β-actin serves as a loading control.

**Supplementary Figure 4**

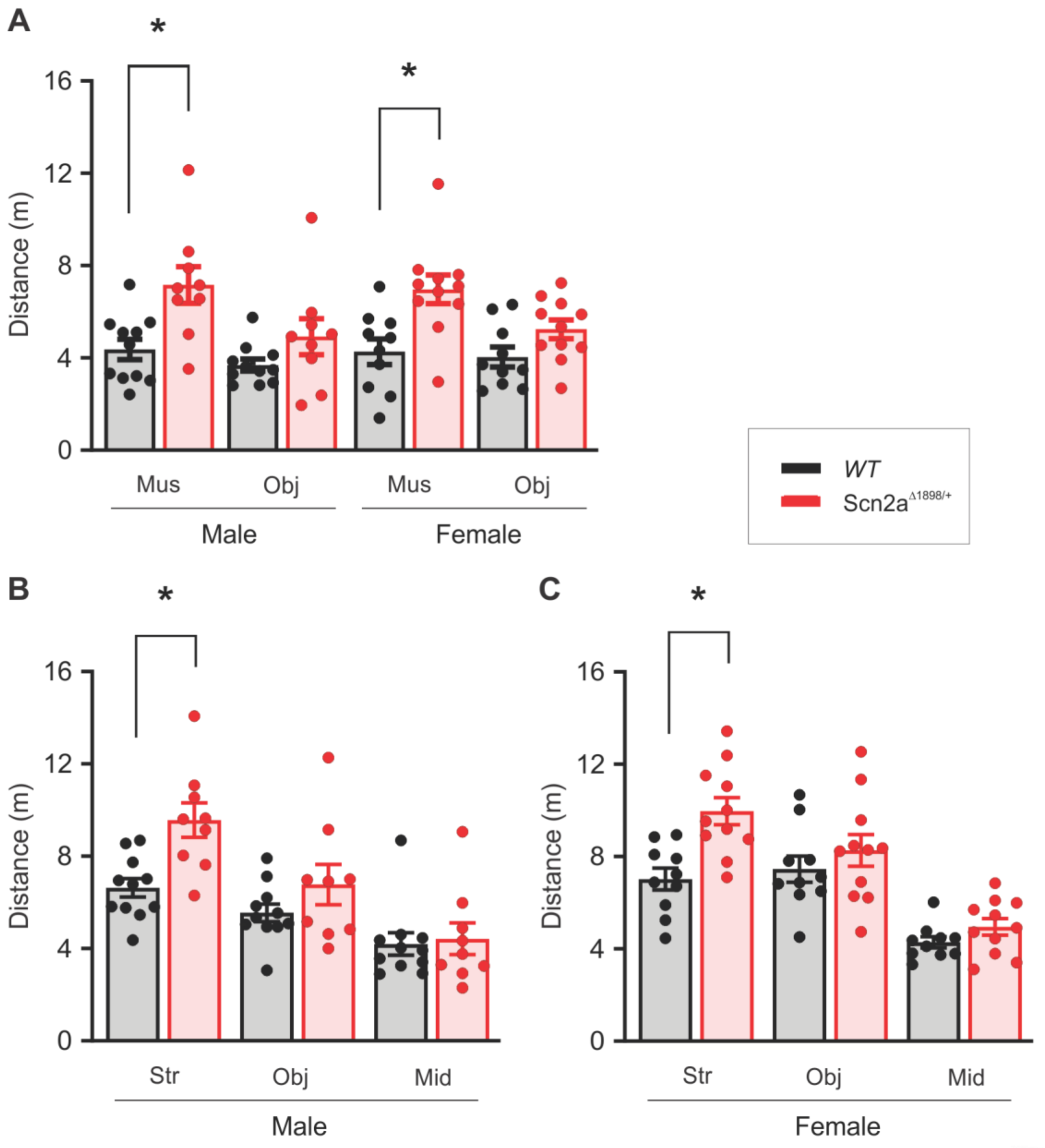


**Supp. Figure 4.** *Scn2a*$^{Δ1898/+}$ mice traveled more in the zone around novel mouse and chamber with novel mouse. ***A***, Distance traveled in the zone around novel mouse (Mus) or object (Obj) (male: *WT*, n=11 and *Scn2a*$^{Δ1898/+}$, n=9; female: *WT*, n=10 and *Scn2a*$^{Δ1898/+}$, n=11). *, unpaired *t* test, t (18)=3.214, *p*=0.0048 for male; t (19)= 3.248, *p*=0.0042 for female. ***B***, ***C***, Distance traveled in the stranger (Str, with novel mouse), object (Obj, with object) and middle (Mid, between stranger and object) chambers. *, unpaired *t* test, t (18)=3.618, *p*=0.002 for male; t (19)= 3.861, *p*=0.001 for female.

**Supplementary Figure 5**

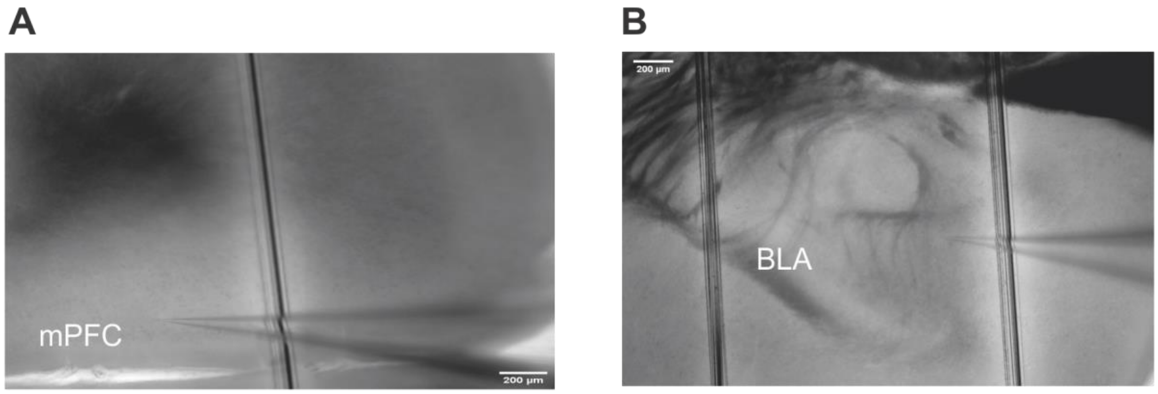


**Supp. Figure 5.** Micrographs showing brain slices with inclusion of mPFC (*A*) or BLA (*B*) from which sEPSCs and sIPSCs were recorded in pyramidal neurons. Scale bar, 200 µm.

**Fig. 1E**

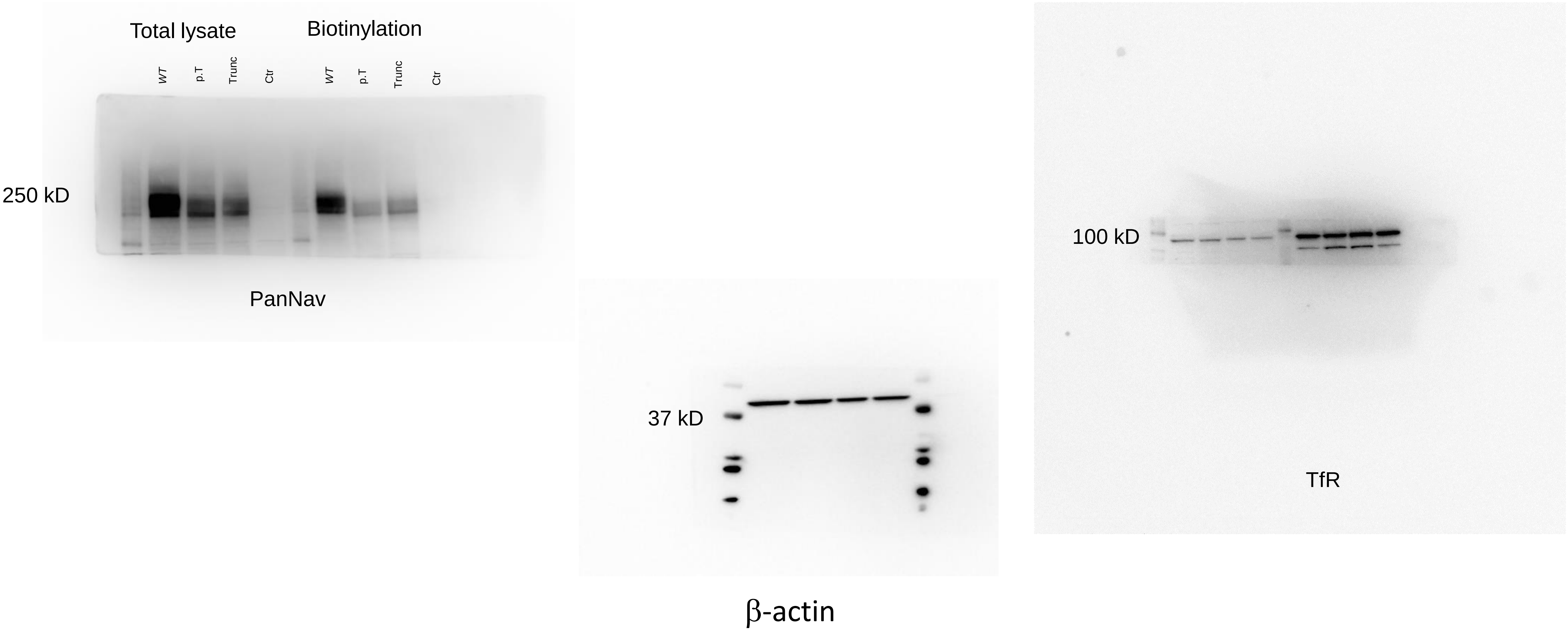
Total lysate
Biotinylation
WT
p.T
Trunc
Ctr
WT
p.T
Trunc
Ctr
250 kD
PanNav
37 kD
β-actin
100 kD
TfR

**Fig. 2A**

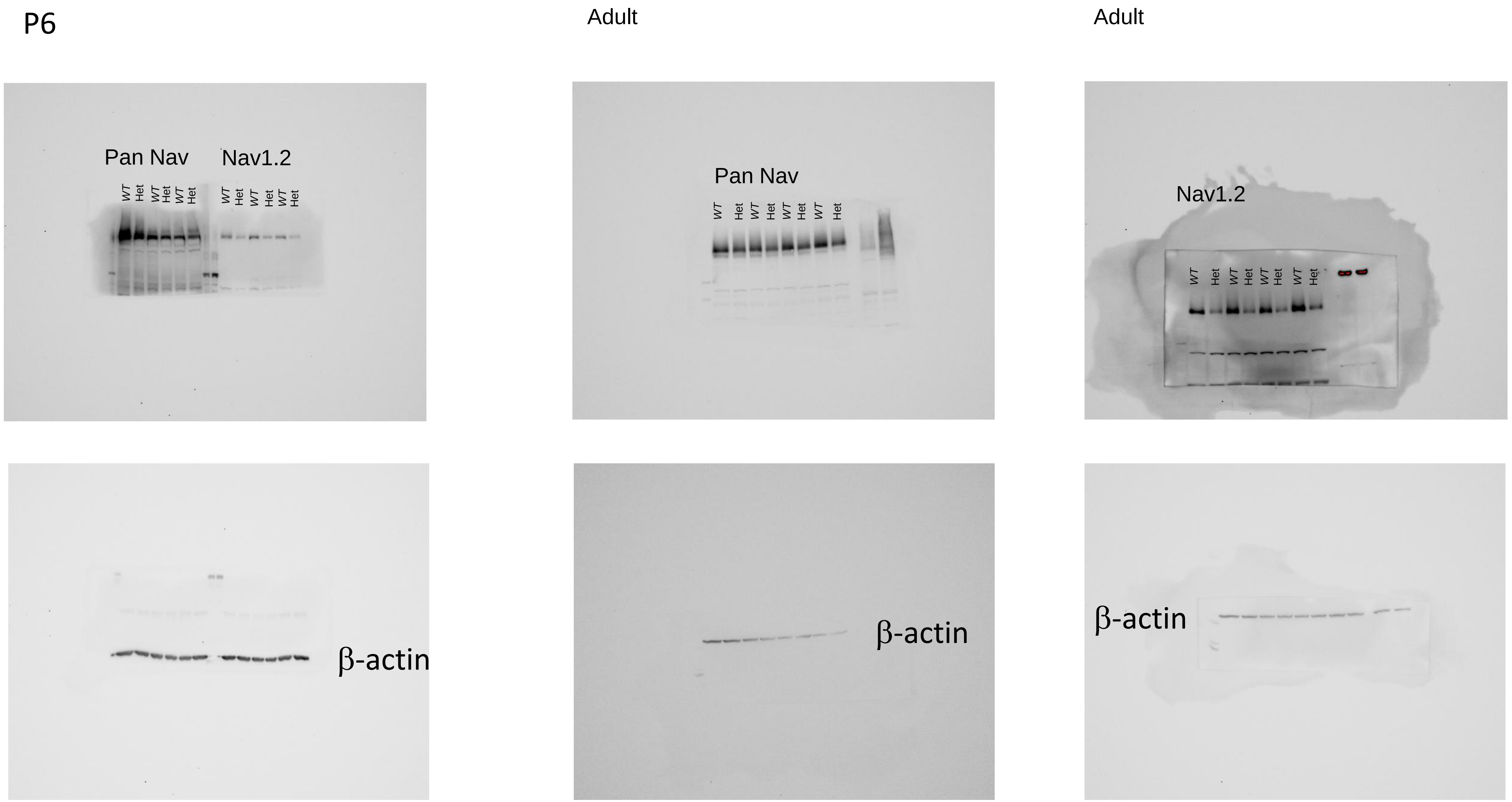
P6
Adult
Adult
Pan Nav
Nav1.2
WT Het WT Het WT Het
WT Het WT Het WT Het
Pan Nav
WT Het WT Het WT Het WT Het
Nav1.2
WT Het WT Het WT Het WT Het
β-actin
β-actin
β-actin

**Supplemental Fig. 3**

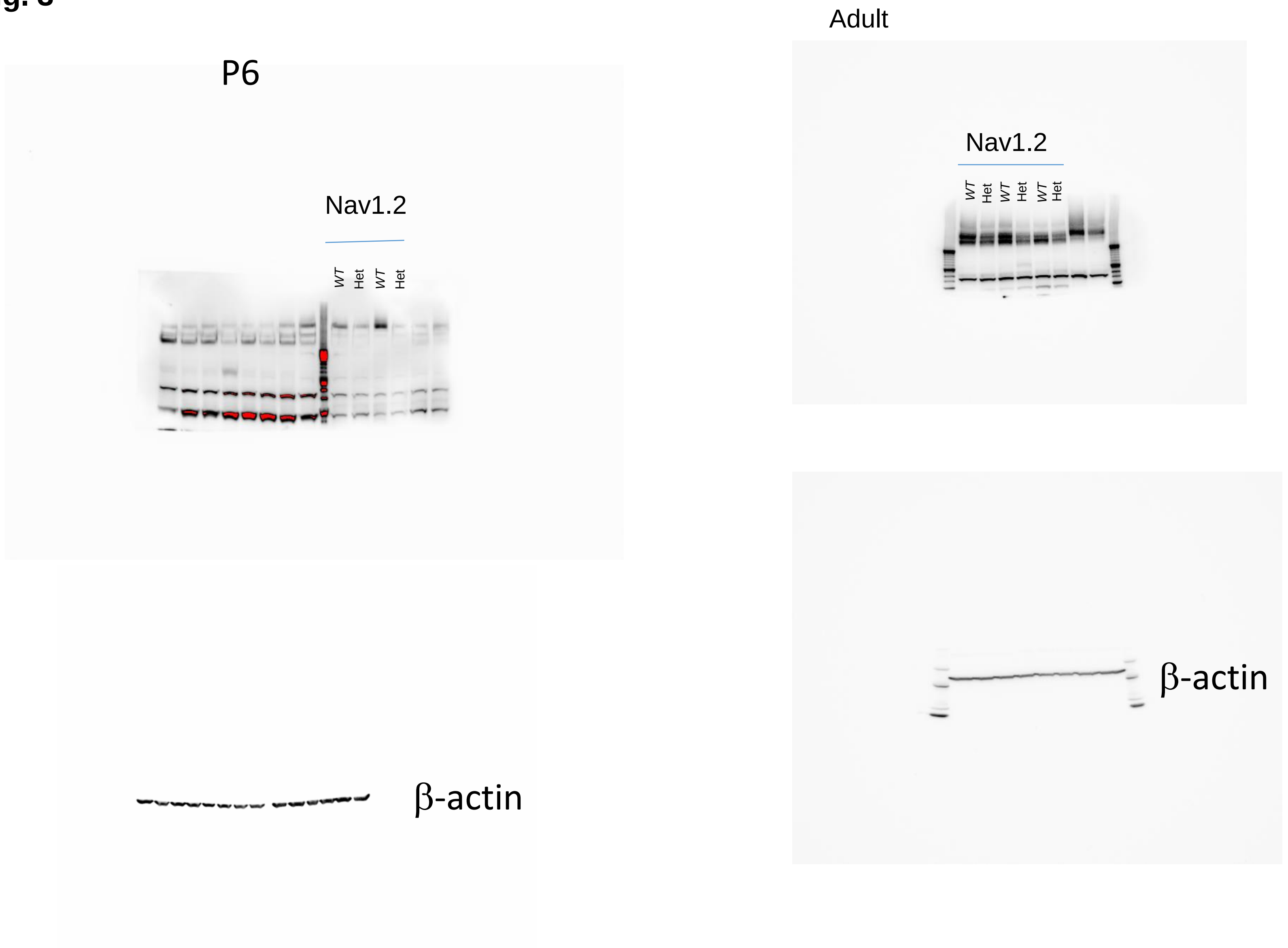

Supplement: Supplemental data [file jciinsight-6-150698-s124.pdf]
